# Nano‐Silver‐Selenium Liquid Dressing Facilitates Treatment of Monkeypox and Prevention of Viral Transmission in a Surrogate Mouse Model

**DOI:** 10.1002/EXP.20240253

**Published:** 2026-05-28

**Authors:** Wei Wang, Mengjun Li, Zining Liu, Jiayin Chen, Ke Liu, Fengfang Wei, Junrui Li, Yixuan Xie, Yushan Jiang, Tyuji Hoshino, Vladislav Victorovich Khrustalev, Minghui Yang, Hua Ma, Ruilin Zhang, Chenguang Shen, Yuhui Liao

**Affiliations:** ^1^ Institute For Engineering Medicine NHC Key Laboratory of Drug Addiction Medicine Kunming Medical University Kunming China; ^2^ BSL‐3 Laboratory (Guangdong) Guangdong Provincial Key Laboratory of Tropical Disease Research School of Public Health Department of Laboratory Medicine Zhujiang Hospital Southern Medical University Guangzhou Guangdong China; ^3^ Key Laboratory of Infectious Diseases Research in South China (Southern Medical University) Ministry of Education Guangzhou Guangdong China; ^4^ The Affiliated Hospital of Putian University Putian Children's Hospital Putian China; ^5^ Hainan Medical College Affiliated Danzhou People's Hospital Danzhou Hainan China; ^6^ Graduate School of Pharmaceutical Sciences Chiba University Chuo‐ku Chiba Japan; ^7^ Department of General Chemistry Belarusian State Medical University Minsk Belarus; ^8^ Advanced Research Institute of Multidisciplinary Science Key Laboratory of Molecular Medicine and Biotherapy Beijing Institute of Technology Beijing China

**Keywords:** antiviral therapy, liquid dressing, monkeypox, nano‐silver‐selenium, transmission blockage

## Abstract

The recent monkeypox (MPOX) outbreak has drawn heightened international concern; however, no effective therapeutic interventions or reliable strategies to prevent its spread are currently available. Here, we developed a nano‐silver (Ag)‐selenium (Se) liquid dressing (lll) by conjugating Ag and Se nanoparticles with a commercial liquid dressing. After administration to the lesion sites, this unique formulation demonstrated highly effective antiviral properties in a surrogate MPOX mouse model (characterised by skin lesions/rashes) induced by vaccinia virus infection. In addition, AgSe@LD showed pronounced anti‐inflammatory activity and accelerated the healing process of virus‐infected cutaneous lesions. Remarkably, AgSe@LD formed a thin film that exhibited excellent adhesion and stability when applied to lesions, while showing resistance to water, alcohol and soapy water washing, thus offering great potential for practical application. This simple and effective liquid dressing represents a major breakthrough in the management of skin infections and provides new ideas for the treatment of MPOX.

## Graphical abstract

There is no effective method to treat this virus and prevent its transmission. To address this, we have developed a nano‐silver‐selenium liquid dressing (AgSe@LD) by combining silver and selenium nanoparticles with commercial liquid dressings. This unique AgSe@LD formulation exhibits good adhesion, stability and toughness and demonstrates efficient antiviral properties against cowpox virus in mouse models, with promising results in reducing inflammatory responses and promoting skin wound healing. Notably, this simple and effective liquid dressing holds great commercial potential and provides new insights for the prevention and treatment of monkeypox.

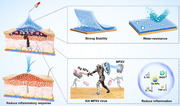

## 1 Introduction

Monkeypox (MPOX) is a zoonotic disease caused by the monkeypox virus (MPXV) [1]. By May 2022, MPOX cases had emerged in numerous countries outside the African region where the disease had not previously been endemic [2], forcing the World Health Organisation to declare the MPOX outbreak a ‘public health emergency of international concern’. As of April 30, 2025, a total of 142,151 confirmed MPOX cases, including 328 deaths, have been reported across 133 countries and territories worldwide [3]. More alarmingly, the number of MPXV infections continues to increase, posing a significant threat to global health and safety. Therefore, it is crucial to develop effective strategies for the treatment and prevention of MPOX.

After an incubation period of 7–14 days, the MPOX patients develop a large quantity of skin rashes containing live, replicating viral particles, which is the primary mode of MPXV transmission [4, 5]. Secretions from these rashes that contain virions may contaminate items such as bed linens, clothing and doorknobs when the patients touch them. Previous studies have shown that MPXV is resistant to desiccation and can survive on the surfaces of the above objects [6, 7], leading to its spread. Thus, interrupting the transmission of MPXV through rashes is critical to controlling the MPOX epidemic [8]. However, there is currently no specific medicine for MPOX in humans [9]. Although previous studies have reported the use of neutralising monoclonal antibodies, antivirals (such as ticovirvir, cidofovir, buunisifovir) and aggregation‐induced‐emission biomaterials to combat MPOX, their effectiveness needs to be further confirmed [10, 11, 12].

Silver nanoparticles (Ag NPs) have been used in the development of antibacterial wound dressings [13, 14, 15, 16, 17] and have shown great potential for antiviral applications. For instance, it has been reported that Ag NPs bind preferentially to the glycoprotein gp120 of human immunodeficiency virus (HIV) [18]. Moreover, Ag NPs may interact with the two disulfide bonds (which are involved in CD4 receptor binding) located in the carboxyl half of the HIV‐1 gp120 glycoprotein [19]. The interaction of Ag^+^ with sulfhydryl groups can cause the reduction of disulfide bonds and lead to protein denaturation, which inhibits the binding between HIV gp120 and the target cell membrane receptors [20]. Additionally, Hu et al. found that Ag NPs could bind to thiol‐containing membrane glycoprotein of HSV‐2, thereby preventing viral internalisation by inhibiting the interactions between glycoproteins and their receptors [21]. Furthermore, Ag NPs have shown the ability to bind to the double‐stranded DNA of hepatitis B virus (HBV), thus inhibiting viral replication and hindering the formation of virus particles [22]. These studies suggested that Ag NPs can target various stages of the viral life cycle, effectively hinder viral proliferation within host cells and exhibit robust antiviral activity [23].

On the other hand, selenium nanoparticles (Se NPs) have gained considerable attention in recent years for their potent antioxidant and anti‐inflammatory properties [24], antimicrobial activities [25], as well as wound‐healing‐promoting capabilities [26, 27, 28]. Of note, Se NPs also have a strong antiviral potential, which acts through various mechanisms, such as up‐regulation of GP × 1 and down‐regulation of Caspase 3 [29]. Studies have indicated that Se NPs can eliminate free radicals and protect DNA from oxidative damage both in vivo and in vitro. For example, they can suppress H1N1 influenza virus–induced apoptosis by inhibiting p53 signalling and the reactive oxygen species (ROS)–mediated AKT pathway [30, 31]. When Se NPs were delivered to HepG2 liver cells infected with HBV, the levels of pro‐inflammatory markers, tumour necrosis factor (TNF) and transforming growth factor, decreased by 1.5‐fold and 1.3‐fold, respectively and the levels of IL‐8 and IL‐2 decreased by 46% and 43%, respectively, indicating that Se NPs exert anti‐HBV‐infection effects by modulating oxidative stress and apoptosis [32].

To date, no studies have reported the application of Ag NPs and Se NPs for targeting *Orthopoxvirus*. Considering that MPXV is a double‐stranded DNA enveloped virus [33], we hypothesised that the combination of Ag NPs and Se NPs could enhance antiviral efficacy through complementary mechanisms. On the one hand, Ag NPs can exert their antiviral effects via a variety of mechanisms, including the decomposition of Ag NPs into Ag^+^, the generation of ROS, direct binding to the viral genome, causing disruption of viral structure and preventing viral proliferation within the host cells [34]. On the other hand, Se NPs possess both anti‐inflammatory and antiviral properties and they can down‐regulate viral loads by promoting chromatin condensation and release of ROS. Moreover, Se NPs can facilitate wound healing through selenomethionine substitution [29]. Therefore, theoretically, AgSe@LD composites have significant antiviral activity and are effective in reducing inflammation and accelerating wound healing. This shows considerable practical implications for the exploration of treatment and prevention strategies for MPOX, particularly in the context of the current pandemic.

**SCHEME 1:**
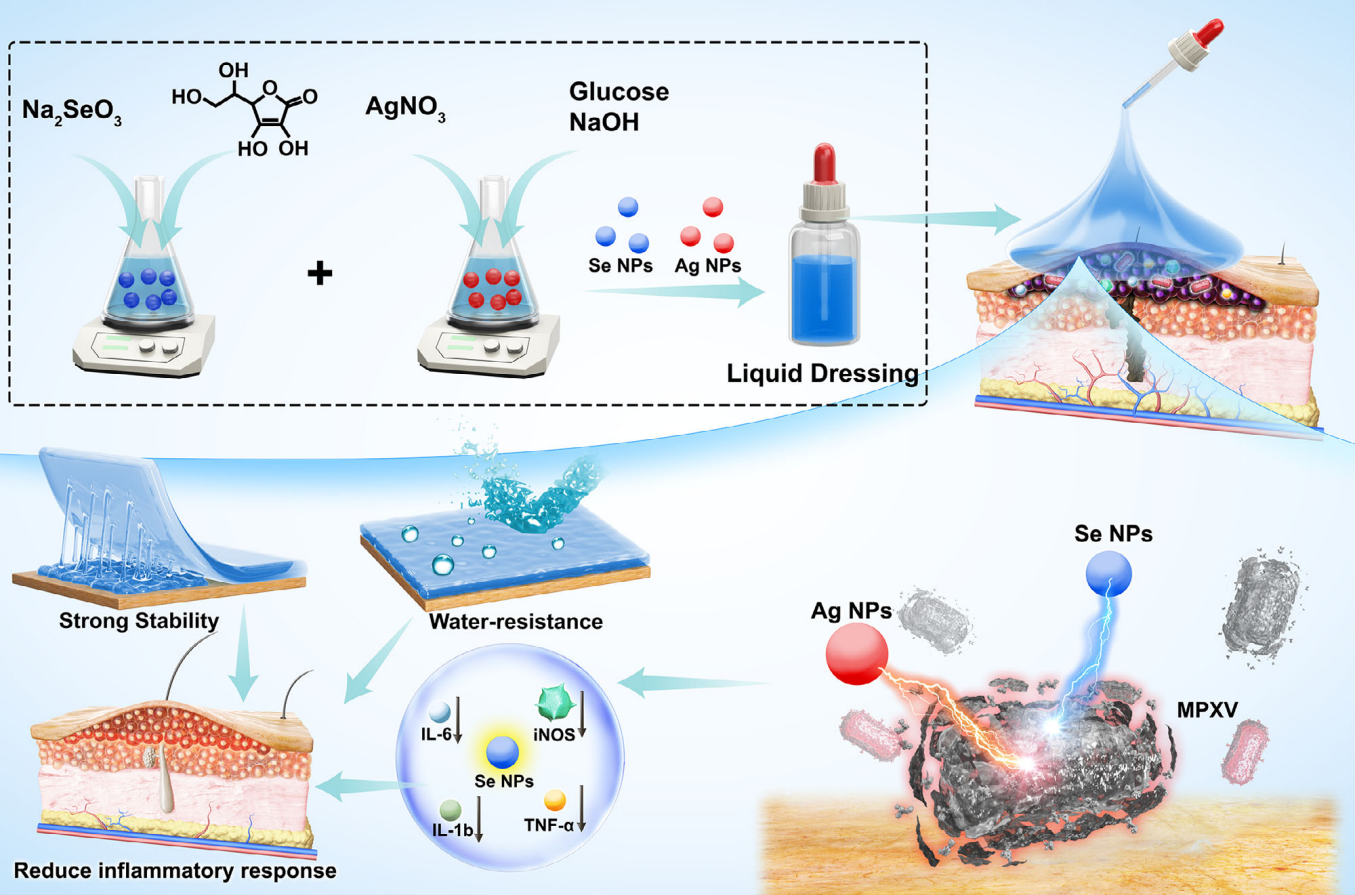
AgSe@LD spray for effective treatment of MPOX and blocking the transmission of MPXV. AgSe@LD, nano‐silver‐selenium liquid dressing; MPOX, monkeypox; MPXV, monkeypox virus; Ag NPs, silver nanoparticles; Se NPs, selenium nanoparticles.

As shown in Scheme 1, Se NPs were synthesised by reducing sodium selenite (Na_2_O_3_Se) using ascorbic acid drops. Simultaneously, we added AgNO_3_ to a mixture of glucose and sodium hydroxide, which was decomposed and reduced under strong magnetic agitation to form Ag NPs. Finally, the two NPs were thoroughly mixed within a commercially available liquid dressing (LD) to prepare AgSe@LD. Concurrently, we established a mouse model mimicking MPOX rash by infecting mice with vaccinia virus via tail scratches. Subsequently, we applied the prepared AgSe@LD to the mouse rashes to evaluate their therapeutic effects in vivo. Typically, the presence of Se NPs in the AgSe@LD effectively reduced inflammation at the lesion site and promoted wound healing. It also prevented the spread of the virus by forming a thin film over the lesions that effectively blocked direct and indirect viral transmission. In addition, this unique strategy shortened the recovery period and healing time of infected animals. These findings indicated that AgSe@LD is highly promising for the treatment and control of MPOX (Scheme 1).

## 2 Experimental Section

### 2.1 Materials

Cell counting kit‐8 (CCK‐8) was purchased from Thermo Fisher Scientific (Waltham, MA, USA). Fetal bovine serum (FBS), Dulbecco's modified Eagle's medium (DMEM), penicillin–streptomycin, trypsin and phosphate‐buffered saline (PBS) were purchased from Gibco. An anti‐vaccinia virus polyclonal antibody (PAB13861) was obtained from Abnova Science and Technology Co., Ltd. DAPI staining solution (cat. KGA1808) were purchased from KeyGEN Biotechnology. Mouse IL‐1β, IL‐6 and TNF‐α enzyme‐linked immunosorbent assay (ELISA) kits were purchased from Jiangsu Meimian Industrial Co., Ltd. (Jiangsu, China). Commercial liquid dressings were purchased from Shandong Qiangshen Pharmaceutical Co., Ltd. All chemicals were used as received without further purification.

### 2.2 Preparation and Characterisations of Ag NPs, Se NPs and AgSe@LD

Synthesis of Ag NPs. First, the reduction solution was prepared by mixing 1 g of D‐glucose, 4 g of NaOH and 400 mL of double‐distilled water (DDW). Subsequently, 0.1 g of AgNO_3_ was dissolved in 100 mL of DDW to obtain a metal precursor solution. Under strong magnetic stirring, an Ag‐containing solution was added to the previously prepared reduction solution. Subsequently, the Ag NPs were successfully prepared. The Ag NPs were washed three times with DDW (8000 rpm, 5 min). Synthesis of Se NPs. The Se NPs were prepared by optimising a previously established protocol. First, sodium selenite was reduced by ascorbic acid [35]. Briefly, Na_2_SeO_3_ was dissolved in DDW to a concentration of 800 µg/mL. Subsequently, ascorbic acid (200 µL, 56.7 mM) was added dropwise to 1800 µL of sodium selenite solution and mixed thoroughly. Se NPs were formed when the colour changed from transparent colourless to transparent red. Synthesis of AgSe@LD. The AgSe@LD was prepared by thoroughly mixing Ag and Se NPs with commercial liquid dressing (LD) (Shandong Qiangshen Pharmaceutical Co. Ltd., Brand: Haishi Hainuo, Number: 10079908965750). The Ag NP and Se NP concentrations were 1 mg/mL. The chemical compositions of both surfaces were verified using X‐ray photoelectron spectroscopy (XPS) and energy‐dispersion X‐ray (EDX) spectroscopy. At the same time, the size and stability of the Ag and Se NPs in the colloidal solution were determined by dynamic light scattering (DLS) and measuring zeta potential and their shapes and distributions were observed by transmission electron microscopy.

### 2.3 AgSe@LD Stability Test

To evaluate the flexibility and mechanical stability of the spray‐coated AgSe@LD film, the AgSe@LD suspension was applied to a mould surface to form a membrane. After drying, the film was carefully peeled off with tweezers and stretched to twice its original length to observe its elasticity. For the adhesion test, the film was applied to the skin surface of the forearm. One edge was lifted with tweezers and an external force was applied to determine whether the opposite side remained adhered to the skin. In the flexibility test, the film was attached to a finger joint and subjected to 90° flexion and extension movements, repeated for 500 high‐intensity bending cycles. The adhesion status and changes in film area were recorded. For the wash resistance test, the film‐adhered skin was sequentially immersed in running water, 75% ethanol and soapy water for one hour each. Any instances of film peeling or dissolution were documented.

In the animal model test, the AgSe@LD film was applied to the dorsal skin of mice. Under free‐moving conditions, images of the application area were captured at 3, 6, 12 and 24 h to evaluate its adhesive durability on dynamic skin surfaces. All human skin adhesion studies were reviewed and approved by the Ethics Committee of Kunming Medical University (Approval No.: KMMU2024MEC287). All animal studies were reviewed and approved by the Experimental Animal Ethics Committee of the Laboratory Animal Centre at Southern Medical University (Approval No.: D202310‐1).

### 2.4 Cell Culture and Viruses

The baby Syrian hamster kidney cell line, BHK‐21; the murine macrophage cell line, RAW 264.7; the mouse epithelioid fibroblast cell line, L929; and a human umbilical vein endothelial cell line were purchased from the American Type Culture Collection (Manassas, VA, USA). All cells were cultured in Roswell Park Memorial Institute 1640 medium or DMEM supplemented with 10% FBS and 1% penicillin–streptomycin at 37°C in a humidified atmosphere with 5% CO_2_. Vaccinia virus strain Tian Tan and GPF‐tagged vaccinia virus strain Tian Tan were propagated and titrated on BHK‐21 cell monolayers. All vaccinia‐virus‐related experiments were performed in a biosafety level II laboratory.

### 2.5 Median Tissue Culture Infective Dose (TCID_50_)

BHK‐21 cells were cultured in a T75 culture flask until reaching 90% confluency, followed by centrifugation at 800 rpm for 5 min to collect and enumerate the cells. Approximately 5 × 10^3^ cells were inoculated into each well of a 96‐well plate with a volume of 100 µL per well and incubated overnight to form a monolayer. Subsequently, 230 µL of DMEM maintenance medium was added to each well of a new 96‐well plate, followed by the addition of 105 µL of vaccinia virus treated with PBS, Ag NPs, or Se NPs into the first well. After thorough mixing, an aliquot of this mixture (105 µL) was transferred to the second well using a fresh tip and mixed again before transferring another aliquot (105 µL) to the third well. This process was repeated for a total dilution factor of 14. Cells in the AgSe@LD group were diluted following the same procedure and applied to the cell plate prior to virus treatment. Each virus sample was added to pre‐seeded wells at a volume of 35 µL per well, with five replicate wells used for each condition. The remaining wells served as blank controls. The plates were incubated in a CO_2_ incubator at 37°C for 1 h before adding an additional volume of DMEM containing 10% FBS (180 µL). Cell pathology was observed on the fourth day and the results were recorded and calculated using the Reed‐Muench method.

### 2.6 Using Vaccinia Virus to Establish a Rash Replacement Model After MPXV Infection

Male BALB/c mice aged 6–8 weeks were purchased from the Southern Medical Experimental Animal Centre, and their use was approved by the ethics committee (Approval No.: D202407‐13). After anaesthetising the mice, a 29‐G injection needle was used to scratch the tails of the mice, followed by the injection of 20 µL of virus with a titer of 1 × 10^6^ into the wound. All experiments were conducted at the BSL‐2 laboratory of the Guangdong Provincial Key Laboratory of Tropical Disease Research. One week later, tail lesions were observed in the mice. Yellow scabs and rash‐like lesions were observed, indicating that the mouse tail‐scratch model (MPOX rash replacement model) was successfully established [36, 37].

### 2.7 In Vivo Antiviral and Anti‐Inflammatory Activity and Prevention of Vaccinia Virus Transmission

Based on the aforementioned criteria, 20 mice were randomly selected and evenly divided into four groups. A tail‐scratch mouse model was established according to the abovementioned method. Six days later, Se NPs, Ag NPs and AgSe@LD were applied to the skin lesions of the mice and the tail lesions of the different groups were photographed every 3 days and observed continuously for 2 weeks. After euthanising the mice by cervical dislocation, tail tissues were collected from each group. Viral titers and necrosis levels at the lesion sites were analysed by quantitative reverse transcription polymerase chain reaction (qRT‐PCR), immunohistochemistry and hematoxylin and eosin (HE) staining. The extent of damage and antigen density were calculated using ImageJ software (National Institutes of Health, Bethesda, MD, USA) to provide a more specific analysis of the results. To further evaluate in vivo antiviral and anti‐inflammatory activity, the collected tail specimens were separated, ground and re‐suspended in 1000 µL of 1× PBS, homogenised using a refrigerated grinder (LUKYM‐I, 70 Hz, 5 min). The samples were then centrifuged at 12,000 rpm for 5 min and the supernatant was collected. Vaccinia virus titers were determined by qRT‐PCR. The primer sequences for qPCR were as follows: vaccinia virus forward, 5′‐ACATCTGGAGAATCCACAACA‐3′; vaccinia virus reverse, 5′‐CATCATCGGTGGTTGATTTA‐3′; vaccinia virus probe, 5′‐FAM‐GAGACTCCGGAACCAAT‐TAMRA‐3′. The levels of pro‐inflammatory cytokines in each group were measured using mouse IL‐6 and IL‐1β precoated ELISA kits to evaluate the in vivo anti‐inflammatory activity of different treatment strategies. Immunohistochemistry and HE staining were used to visually determine the viral antigen density and the level of tissue necrosis, respectively. Image J was used to calculate the scores. Subsequently, to evaluate the effect of different treatment strategies on blocking virus transmission in vivo, equal amounts of the collected samples from each group were added to 1 mL of 1× PBS and then ground in a frozen grinder (LUKYM‐I, 70 Hz, 5 min), centrifuged at 12,000 rpm for 5 min to obtain the supernatant. The virus was inoculated into scratches on the tails of normal mice and the scratches were observed and recorded using a camera for 12 days. The results were analysed using Image J software and GraphPad Prism 7.0 (GraphPad, San Diego, CA, USA). The pro‐inflammatory cytokines IL‐6 and IL‐1β were quantified using ELISA kits. Immunohistochemical staining, HE staining and Masson's trichrome staining were also used to quantify viral particles and inflammatory factors in the collected tail‐diseased tissues and to evaluate the density of viral antigens and the level of tissue necrosis. Image J was used to calculate the scores.

### 2.8 Biosafety Assessment

To evaluate the biocompatibility of the different therapeutics in vitro and in vivo, Se NPs, Ag NPs and AgSe@LD membranes were incubated with BHK‐21 cells for 48 h and their proliferation and viability were determined using a CCK‐8 assay (Beyotime Biotechnology, Nantong, China). Simultaneously, a haemolysis experiment was conducted, in which the patients were exposed to a certain concentration of AgSe@LD and continuously observed for 48 h. The haemolysis rates were recorded at 0, 6, 12, 24 and 48 h. Healthy male BALB/c mice (6–8 weeks old) were exposed to Se NPs, Ag NPs, or AgSe@LD for 12 days and their body weights were monitored as a measure of in vivo toxicity. On the 12th day, blood samples were collected from the mice to assess the liver function measures of albumin (ALB), aspartate aminotransferase (AST) and alanine aminotransferase (ALT) and renal function by measuring creatinine (CR) and blood urea nitrogen (BUN). The skin and penises were collected from each group of mice for histological analysis and cytokine detection.

### 2.9 Statistical Analysis

The data are presented as the mean ± standard deviation (SD) from a minimum of three independent experiments. Group differences were analysed using a paired Student's t‐test and one‐way analysis of variance, followed by Tukey's post‐hoc test (GraphPad Prism 7.0). Statistical significance was set at *p* < 0.05.

## 3 Results

### 3.1 Synthesis and Characterisation of Ag NPs, Se NPs and AgSe@LD

**FIGURE 1:**
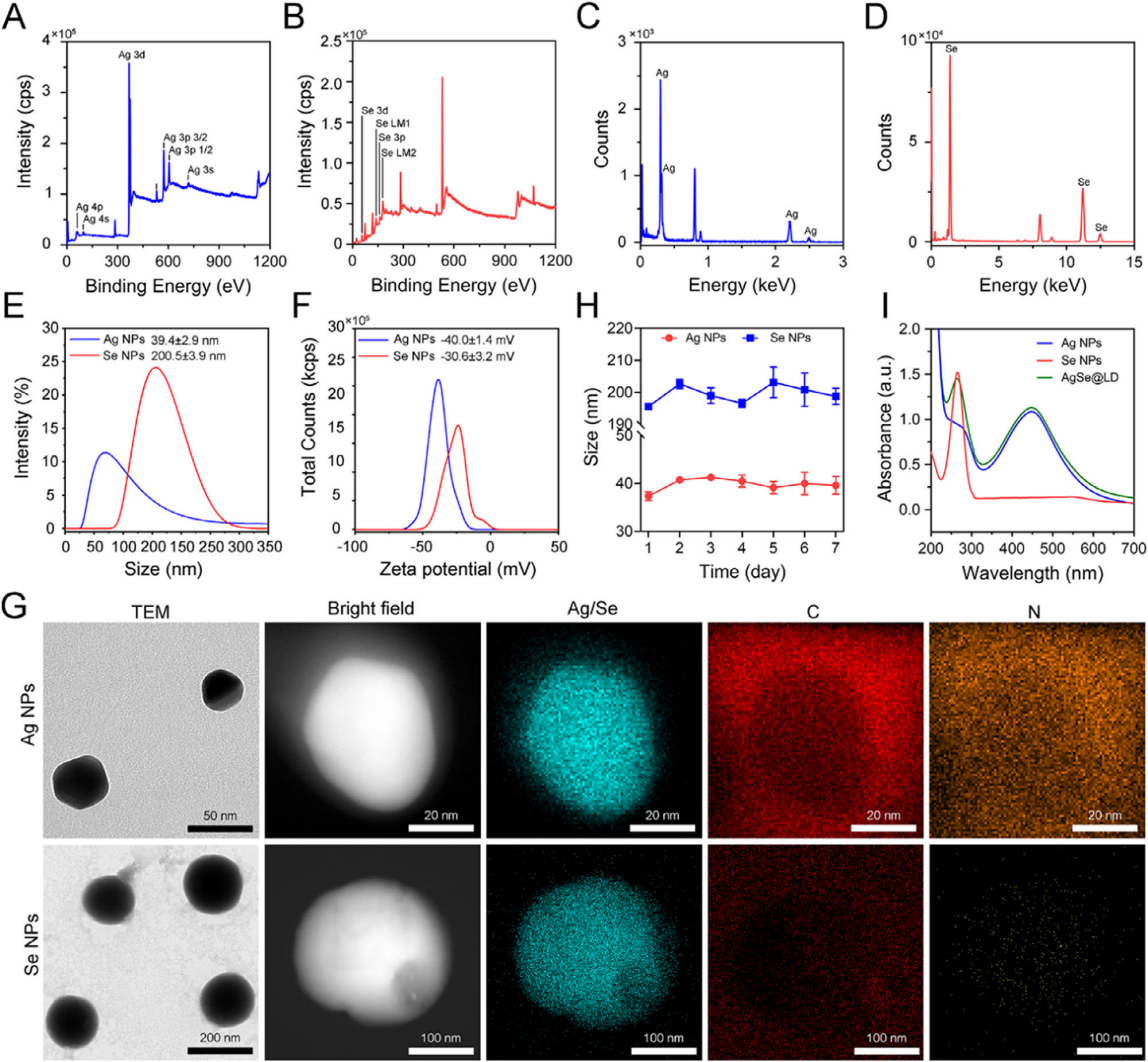
Synthesis and characterisation of Ag NPs, Se NPs and AgSe@LD. (A, B) X‐ray photoelectron spectroscopy analysis of silver nanoparticles (Ag NPs) (A) and selenium nanoparticles (Se NPs) (B). Elemental analysis of Ag NPs (C) and Se NPs (D) using energy‐dispersive X‐ray spectroscopy. (E, F) Hydrodynamic size (E) and zeta potential (F). (G) Transmission electron microscope (TEM) images and elemental analysis of Ag NPs and Se NPs. (H) The diameters of both nanoparticles were monitored separately in PBS. Data are the mean ± standard deviation (SD; *n* = 3). (I) Ultraviolet‐visible absorption spectra of Ag NPs, Se NPs and nano‐silver‐selenium liquid dressing (AgSe@LD).

In this study, Ag NPs, Se NPs and AgSe@LD were successfully synthesised. First, the chemical composition of the surface elements of the Ag and Se NPs was determined by XPS (Figures 1A,B and S1) and the results indicated that the two nanoparticles had distinct dominant peaks. In Ag NPs, the peak values corresponding to the C, H, Ag, N and O elements mainly occurred at 50–60 eV, 95–100 eV, 372–378 eV, 580–590 eV and 600–610 eV, respectively. Among these peaks, the dominant peak was found at 372–378 eV. However, peaks of Se NPs were detected at 50–60 eV, 130–140 eV, 160–165 eV and 176–180 eV, with a prominent peak around 300 eV, which is consistent with previous reports [38, 39]. These findings suggest that the two nanoparticles have different chemical properties and surface states. In addition, EDX spectra (Figure 1C,D) showed that the characteristic absorption peaks of Ag NPs were 0.25, 0.3, 2.2 and 2.5 keV, respectively and those of Se NPs were 1.37, 11.22 and 12.49 keV, respectively [40, 41], indicating that the prepared nanoparticles were based on Ag and Se, respectively and possessed different atomic structures. As shown in Figure 1E, DLS was employed to determine the sizes of the Ag and Se NPs in the colloidal solution. The particle sizes of the prepared Se NPs and Ag NPs were 200.5 ± 3.9 nm and 39.4 ± 2.9 nm, respectively. Notably, the Se NPs were larger than the Ag NPs, which may be attributed to the distinct synthesis methods utilised. Moreover, both nanoparticles exhibited low dispersion indices, indicating their homogeneous distribution within the solution. Furthermore, zeta potential measurements revealed surface charges of −40.0 ± 1.4 mV for Ag NPs and −30.6 ± 3.2 mV for Se NPs (Figure 1F), which is consistent with the findings of previous studies and suggests an abundance of charges along with strong repulsion forces contributing to the stability of these particles. To further validate the sizes, morphology and composition of the biosynthesised Ag and Se NPs (Figures 1G, S2 and S3), transmission and scanning electron microscopy analyses were performed and revealed a spherical shape with high dispersity for Ag NPs, while independently distributed spherical Se NPs displayed good dispersion across the field of view. In addition, no significant change in the size of Ag NPs and Se NPs were observed during a 7‐day monitoring period, indicating good stability of these nanoparticles (Figure 1H). In addition, we examined their ultraviolet‐visible (UV‐vis) absorption spectra and found that AgSe@LD produced characteristic absorption peaks of Ag NPs and Se NPs in the visible light region (Figure 1I), suggesting that the synthesised Ag NPs and Se NPs complexes were relatively pure.

### 3.2 Performance of AgSe@LD

**FIGURE 2:**
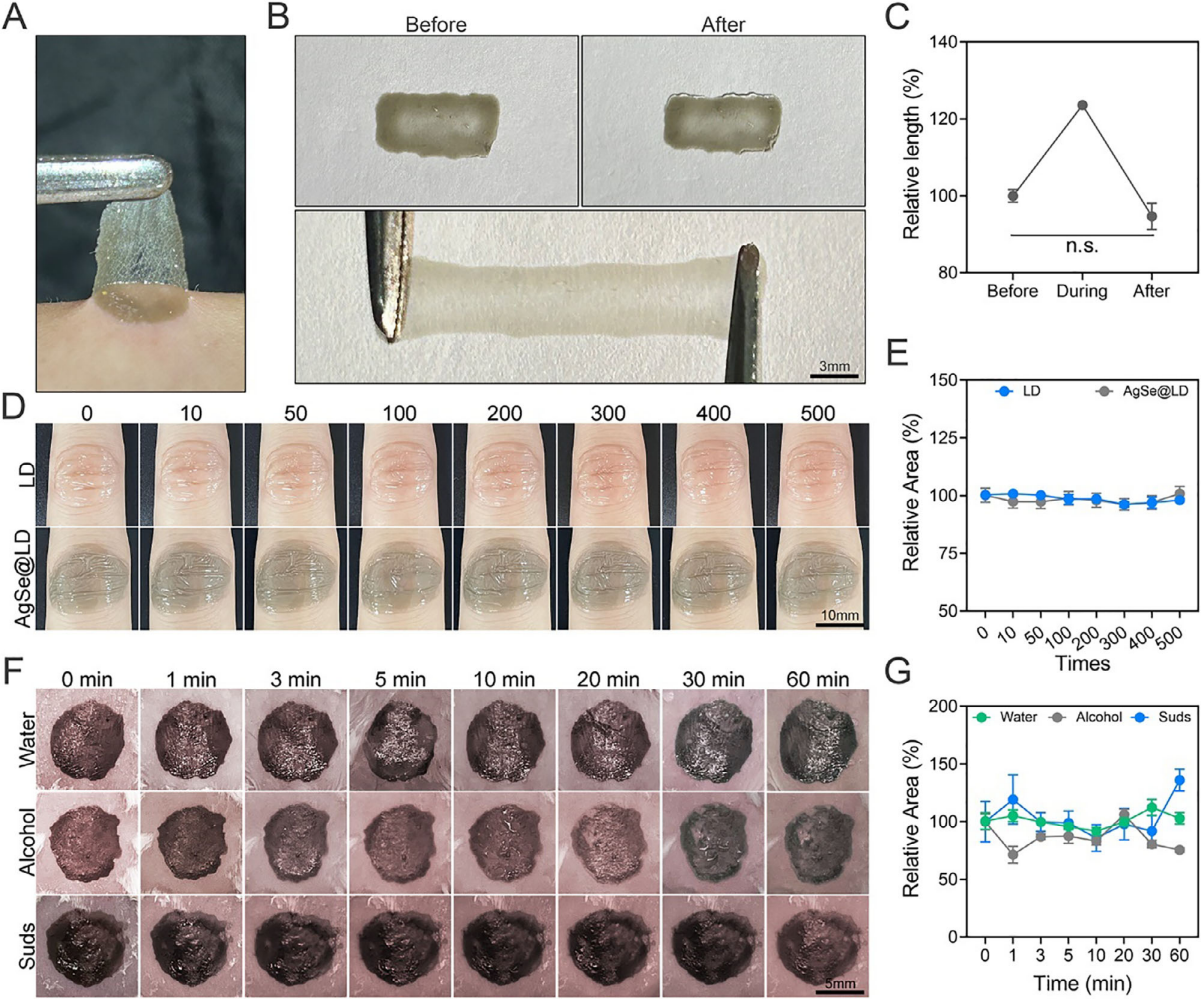
Adhesion, stability and toughness of AgSe@LD. (A) Images of adhesion of the AgSe@LD to skin. (B, C) Images and statistical analysis of the relative length of AgSe@LD before, during and after stretching. Scale bar = 3 mm. The data indicate the mean ± SD (*n* = 3). (D, E) Images and statistical analysis of the relative area of the LD and AgSe@LD after flexion and extension of the finger joint for different times. Scale bar = 10 mm. The mean ± SD (*n* = 3) is shown. (F, G) Images and statistical analysis of the relative area of AgSe@LD with continuously flowing pure water, 75% alcohol and soapy water at different times. Scale bar = 5 mm. Data indicate the mean ± SD (*n* = 3). p* < 0.05, **p < 0.01, ***p < 0.001.

Good adhesion, stability and toughness are critical properties for wound dressings. To evaluate these characteristics of the AgSe@LD, the dressing was first applied to the arm and then tested for adhesion by picking it up with tweezers. After testing adhesion, the dressing was further evaluated for stability and toughness to ensure that it met the requirements for an effective wound dressing. The adhesion, stability and toughness of the AgSe@LD were evaluated using several tests. In the first test, the dressing was applied to the skin and adhesion was tested by lifting it with tweezers. The results showed that the skin was lifted by force (Figure 2A). This indicated that AgSe@LD possesses excellent adhesion properties. Furthermore, when we subjected AgSe@LD to a 3‐fold extension using tweezers, it exhibited no significant deformation before and after stretching (Figure 2B,C). This indicated that the AgSe@LD maintained high stability under external forces. Subsequently, a commercial liquid dressing (LD) and the prepared AgSe@LD were dropped into the phalanx joint to test their adhesion and toughness. As depicted in Figure 2D, the LD and AgSe@LD were applied to finger joints and subjected to repeated flexion and extension. Even after 500 consecutive bends and stretches, the AgSe@LD remained intact. The tensile strength was similar to that of the LD, whereas the area showed minimal change (Figure 2E), indicating that the inclusion of Ag and Se NPs did not compromise the toughness of the LD. Furthermore, when applied to the back skin of mice, AgSe@LD demonstrated no shedding or alterations in size over 24 h (Figure S4). These findings suggested that its ability to form a film on skin lesions with standing forces from the surrounding tissues, exhibiting excellent adhesion and durability. Another important property of wound dressings is their stability, which includes resistance to washing and exposure to different liquids. To test the stability of AgSe@LD under various liquid conditions, we continuously washed the dressing in flowing pure water, 75% alcohol and soapy water for 1 h and observed any changes (Figure 2F). AgSe@LD formed stable films on the skin surface without separation, solidification, or dissolution. The dressing area did not change significantly at the different time points, indicating good stability (Figure 2G). These findings demonstrate that AgSe@LD can effectively cope with changes in the external environment, thereby ensuring a better therapeutic effect. It remained stably attached to the skin even when exposed to different liquids and maintained its adhesion and durability, making it suitable for use as a wound dressing.

### 3.3 Antiviral and Anti‐Inflammatory Activities of AgSe@LD in vitro

**FIGURE 3:**
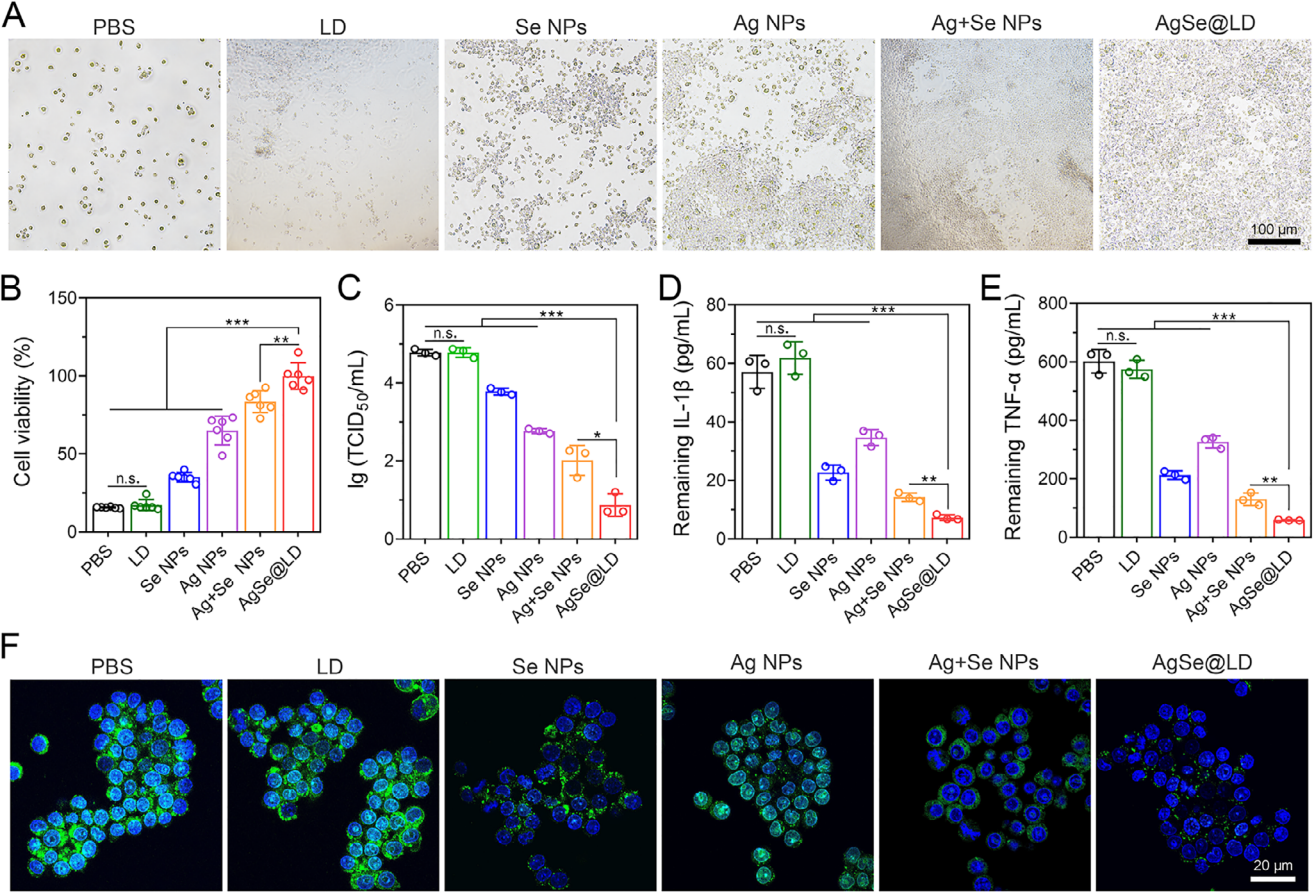
Antiviral and anti‐inflammatory activities of AgSe@LD in vitro. (A, B) Photographs and cell viability analysis of BHK‐21 cells after different treatments, showing the mean ± SD (*n* = 6). (C) Antiviral efficacy assessment (TCID_50_) after different treatments. Data indicate the mean ± SD (*n* = 3). (D–F) Enzyme‐linked immunosorbent assays were used to detect the levels of various cytokines in the cell supernatant after different treatments. The data presented are the mean ± SD (*n* = 3). (F) Confocal image and quantitative analysis of iNOS after different treatments. Blue: DAPI, nuclei. Green: iNOS. Scale bar, 20 µm. Data were analysed by one‐way analysis of variance. *p < 0.05, **p < 0.01, ***p < 0.001.

Referring to the methodology of previous studies [37, 42, 43], we comprehensively evaluated the efficacy of LD, Se NPs, Ag NPs, Ag+Se NPs and AgSe@LD in antiviral infections and alleviation of inflammation in vitro. BHK‐21 cells were infected at a multiplicity of infection (MOI) of 0.5. Following a 1‐hour incubation of the virus with each treatment, the cells were subsequently infected. On day 4 after infection, the cells were fixed and observed under a bright‐field microscope (Figure 3A). Significant changes in cell morphology occurred in the PBS and LD groups, with almost complete cell death. Cytopathic effects were observed in both the Se NP and Ag NP groups; however, the degree of cytopathic effects was relatively low in the Ag NP group, suggesting a more pronounced antiviral effect than Se NPs alone. This result suggests that Ag NPs have stronger antiviral infection efficacy than Se NPs alone. In contrast, the cells in the Ag+Se NPs and AgSe@LD groups showed minimal pathological changes, highlighting their significant synergistic antiviral effects. Compared with PBS and LD control groups, Se NP and Ag NP groups showed different degrees of cell viability (Figure 3B), which was attributed to their strong cytoprotective mechanisms [44]. Notably, cell survival was significantly increased in the Ag+Se NPs and AgSe@LD groups, with the most pronounced effect in the AgSe@LD group, highlighting its potent antiviral activity. In addition, we performed TCID50 analysis to quantify the number of virus particles remaining after treatment. As shown in Figure 3C, all four treatment groups exhibited varying degrees of virucidal activity compared to the PBS and LD controls. The Se NPs group showed a modest reduction in viral load, suggesting potential antiviral and anti‐inflammatory properties [45, 46, 47]. The Ag NPs group also demonstrated a slight decrease in viral load, attributed to the extensive antiviral activity of Ag NPs [48, 49, 50, 51], reaffirming their efficacy against vaccinia virus. Importantly, the Ag+Se NPs and AgSe@LD groups showed significantly lower viral loads, which were attributed to the synergistic effect of Ag NPs and Se NPs. Notably, the AgSe@LD group showed the most significant effect.

Studies have demonstrated that in human MPXV infections, irrespective of disease severity, there is an elevation in the levels of numerous cytokines, such as IL‐1β, IL‐6 and TNF‐α, following infection. Moreover, severely ill patients exhibit higher levels of cytokines than those with milder disease [52]. Consequently, MPXV infection triggers a robust inflammatory response and antiviral drugs aimed at eradicating the virus should also mitigate the body's inflammatory response. Previous studies have demonstrated that Se NPs can decrease the levels of IL‐6 and TNF‐α, while Ag NPs can hinder the production of pro‐inflammatory cytokines, such as TNF‐α and IL‐6, in macrophages [53, 54]. To confirm the anti‐inflammatory effects, the culture medium was collected after treatment. ELISAs were utilised to determine the levels of pro‐inflammatory cytokines (Figure 3D,E), including IL‐1β and TNF‐α. The results indicated that the levels in the Se NP and Ag NP groups were moderately reduced compared to those in the PBS and LD groups. The levels of IL‐1β and TNF‐α in Ag+Se NPs groups and AgSe@LD groups were significantly decreased and the levels in the AgSe@LD group were the most significantly decreased. Additionally, various studies have shown that iNOS expression levels are elevated in patients infected with different pathogens (such as SARS‐CoV‐2, influenza viruses and MPXV), indicating a close correlation between the upregulation of iNOS and the inflammatory response [55]. Therefore, iNOS levels were detected in macrophages in different treatment groups after virus co‐incubation (Figure 3F). As expected, iNOS expression was significantly up‐regulated in the LD and PBS groups, moderately up‐regulated in the Se NP and Ag NP groups and slightly up‐regulated in the Ag+Se NPs and AgSe@LD group. These results collectively indicated that both Se NPs and Ag NPs have anti‐inflammatory activities to a certain extent in vitro. AgSe@LD inhibited the production of pro‐inflammatory cytokines in macrophages and effectively reduced the secretion of inflammatory factors owing to the synergistic effects of the nanoparticles, thus exhibiting effective anti‐inflammatory activity. Therefore, AgSe@LD not only effectively kills viruses but also plays a highly effective antiviral role in reducing cell damage and inflammation.

### 3.4 Antiviral Activity of Se NPs, Ag NPs and AgSe@LD in vivo

**FIGURE 4:**
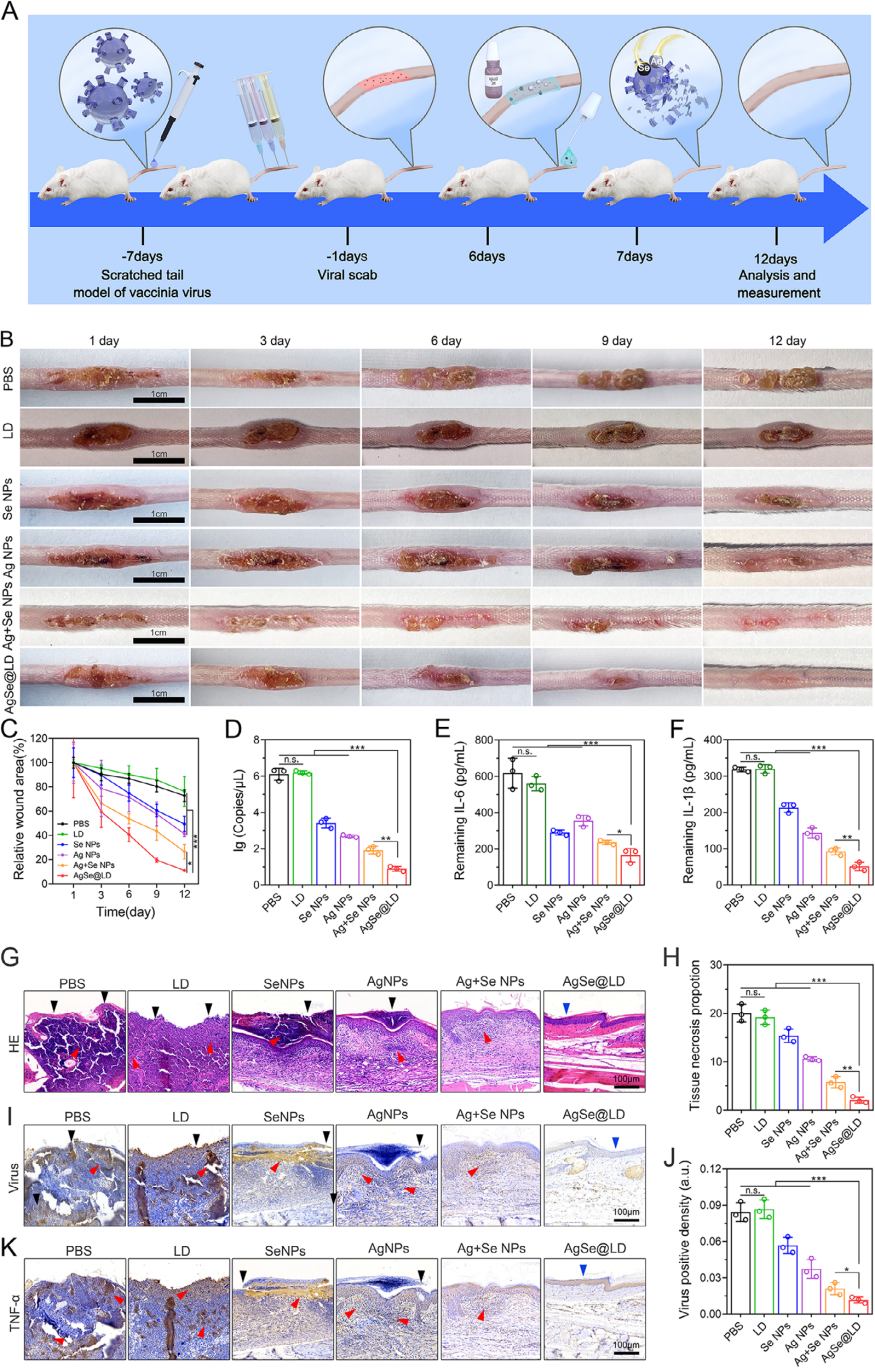
Antiviral activity of LD, Se NPs, Ag NPs, Ag+Se NPs and AgSe@LD in vivo. (A) The establishment of an MPOX rash surrogate model and schematic diagram of different treatments. (B) Differences in injury changes among treatment groups. (scale = 1 cm). (C) Quantitative analysis of tail lesions in different treatment groups using ImageJ software. (D) Viral load at the affected site in different treatment groups. (E, F) Detection of pro‐inflammatory factors (IL‐6 and IL‐1β) in tail lesions after different treatments. (G, H) Quantitative analysis of hematoxylin and eosin (HE) staining and damage area at the lesion site in different treatment groups (scale = 100 µm). (I, J) Immunohistochemical staining and quantitative analysis of virus antigen in different treatment groups (scale = 100 µm). (K) Immunohistochemical staining of TNF‐α in tail lesions of mice in different treatment groups. Data indicate the mean ± SD (*n* = 3). Data were analysed by one‐way analysis of variance. *p < 0.05, **p < 0.01, ***p < 0.001.

To further investigate the in vivo antiviral activity of LD, Se NPs, Ag NPs, Ag + Se NPs and AgSe@LD, we employed a mouse tail scratch model to simulate skin lesions following MPXV infection. Following the infection, large yellow scabs developed at the wound site, accompanied by significant redness, swelling and blistering, indicating robust viral replication and severe inflammation. By Day 9, the redness and swelling in the tail were significantly reduced, the blisters diminished in size and the scabs gradually enlarged before eventually falling off, facilitating wound healing. This successful simulation illustrated the progression of skin lesions post‐MPXV infection. As depicted in Figure 4A, vaccinia virus was injected into the scratch on the tail of a mouse. On day 6, the lesions were treated with LD, Se NPs, Ag NPs, Ag + Se NPs, or AgSe@LD and the subsequent changes were observed and recorded. As shown in Figure 4B, compared to the PBS and LD groups, the four treatment groups exhibited varying degrees of protection. Notably, the Se NPs group demonstrated a slightly shorter healing period, likely due to its anti‐inflammatory and antiviral effects. The Ag NPs group exhibited moderate skin healing, with an accelerated healing rate, possibly attributable to the ability of Ag NPs to inhibit viral replication, reduce inflammation and minimise tissue damage. Mice treated with Ag + Se NPs and AgSe@LD showed rapid recovery, with the latter group recovering the fastest; scabs fell off by day 9 and wounds were nearly completely healed by day 12. This rapid recovery may be due to the aggregation of AgSe@LD within the lesions, resulting in increased local concentration and prolonged action time. These findings suggest that AgSe@LD may synergistically combine the benefits of inhibiting viral replication and reducing inflammatory responses, thereby promoting effective recovery and shortening the healing period. Quantitative analysis of the lesion area in the mouse rash is presented in Figure 4C. In the PBS and LD groups, the lesion area initially increased before decreasing, likely due to extensive viral replication leading to severe inflammatory responses and rash formation. However, since vaccinia is a self‐limiting disease, the lesion area gradually diminished until recovery. The Se NPs and Ag NPs groups moderately promoted wound healing and scar tissue formation, but neither treatment alone completely eliminated live virus. In contrast, the Ag + Se NPs and AgSe@LD groups demonstrated faster recovery and more pronounced effects, with the AgSe@LD group exhibiting the most significant impact.

Previous studies have demonstrated that Se NPs exhibit significant antiviral activity and can serve as antioxidants in normal tissues and cells. This can help regulate the oxidative stress response of immune cells, effectively controlling apoptosis to achieve an anti‐infection effect [56, 57]. Ag NPs also act as antiviral agents by preventing virus entry, replication and release [58]. To further evaluate the antiviral effects of different treatment groups, tail specimens from infected mice of the same weight were collected on the sixth day after treatment. These specimens were resuspended in the same volume of PBS and the supernatants were collected by centrifugation of a homogenate generated using a refrigerated grinder. As depicted in Figure 4D, compared with the PBS and LD groups, the viral loads of the four treatment groups decreased to varying degrees. Both the Se NP and Ag NP groups exhibited a moderate decrease in viral load, indicating a certain degree of in vivo antiviral effect, which is consistent with the findings of previous studies. While the Ag+Se NPs group and the AgSe@LD group had lower viral loads, it is worth noting that the mice treated with AgSe@LD had the lowest viral loads, indicating the most significant antiviral effects in *vivo*. Notably, the viral load was lowest in the mice treated with AgSe@LD, indicating the most significant in vivo antiviral effects. This enhanced efficacy may be attributed to the combined antiviral mechanisms of the two compounds, resulting in a synergistic antiviral effect in vivo. Additionally, the incorporation of LD facilitates the formation of a stable film at the lesion sites, thereby prolonging the duration of action. Additionally, the anti‐inflammatory activity of the different treatment groups was studied by detecting inflammatory factors in the homogenates of diseased tail tissue. As shown in Figure 4E,F, levels of the pro‐inflammatory factors, IL‐6 and IL‐1β, were observed to be elevated in the PBS group, suggesting that vaccinia virus can induce significant inflammation in the affected area of mice, potentially contributing to the observed redness, swelling and abscess. Similarly, the same phenomenon was observed in the LD group. Whereas the Se, Ag, Ag+Se NPs and AgSe@LD groups exhibited varying degrees of reduction in these parameters. The Se NP group demonstrated a moderate decrease, as previous studies have indicated that Se NPs can decrease the expression levels of pro‐inflammatory factors and markers, such as TNF‐α, IFN‐γ, showcasing robust anti‐inflammatory properties [59]. Similarly, the Ag NP group also displayed a reduction in the levels of these factors, likely due to their ability to inhibit the expression of pro‐inflammatory cytokines, such as TNF‐α and IL‐1, in macrophages [60]. In addition, the Ag+Se NPs and AgSe@LD groups showed a significant reduction in levels of proinflammatory factors, with the latter having a more pronounced effect, emphasising its powerful anti‐inflammatory activity, which may be due to increased regulation of proinflammatory factors that enhanced its anti‐inflammatory efficacy over and above that of the individual ingredients.

After the collection of diseased tail tissues, HE staining was performed (Figure 4G,H). The results revealed significant inflammatory infiltration and extensive tissue necrosis in the tail tissues of the PBS and LD groups, indicating the presence of live virus, which may facilitate moderate to severe viral transmission. In contrast, the Se NP and Ag NP groups exhibited only mild inflammatory infiltration and necrosis. Notably, HE staining demonstrated the least pathological damage in the Ag+Se NPs and AgSe@LD groups, with the latter group showing almost no damage. Concurrently, Masson staining (Figure S6) indicated extensive collagen fibre hyperplasia and rupture in the affected tail tissues of the PBS and LD groups, accompanied by pronounced muscle fibre morphology. Conversely, only minor localised changes were observed in the Se NP and Ag NP groups. Consistent with the HE results, the AgSe@LD group exhibited minimal alterations in the number and morphology of collagen fibres. Furthermore, immunohistochemical staining was conducted, as illustrated in Figures 4I,J. Particles deposited in the PBS and LD groups displayed a high density of viral antigens, indicating ongoing viral replication in the affected areas post‐infection. In contrast, particles deposited in the Se NP, Ag NP, Ag+Se NPs and AgSe@LD groups showed a progressive decrease in viral antigen density. Additionally, immunohistochemical staining for TNF‐α and INF‐γ revealed strong nucleo‐positive and diffuse expression in both the PBS and LD groups (Figures 4K and S5), indicating substantial viral replication in the lesions of infected mice, which resulted in a pronounced inflammatory response. Moderate expression was observed in the Se NP and Ag NP groups, suggesting partial viral clearance. However, expression was either absent or weakly expressed in the AgSe@LD group, indicating the efficacy of AgSe@LD in eliminating live virus and mitigating inflammation in the affected area. These findings suggested that the tail lesions of the AgSe@LD‐treated mice contained almost no live virus and did not lead to virus transmission. Therefore, AgSe@LD exhibited potential for the treatment of MPXV and has broad application prospects.

### 3.5 Efficacy of Se NPs, Ag NPs and AgSe@LD in Blocking Virus Transmission in vivo

**FIGURE 5:**
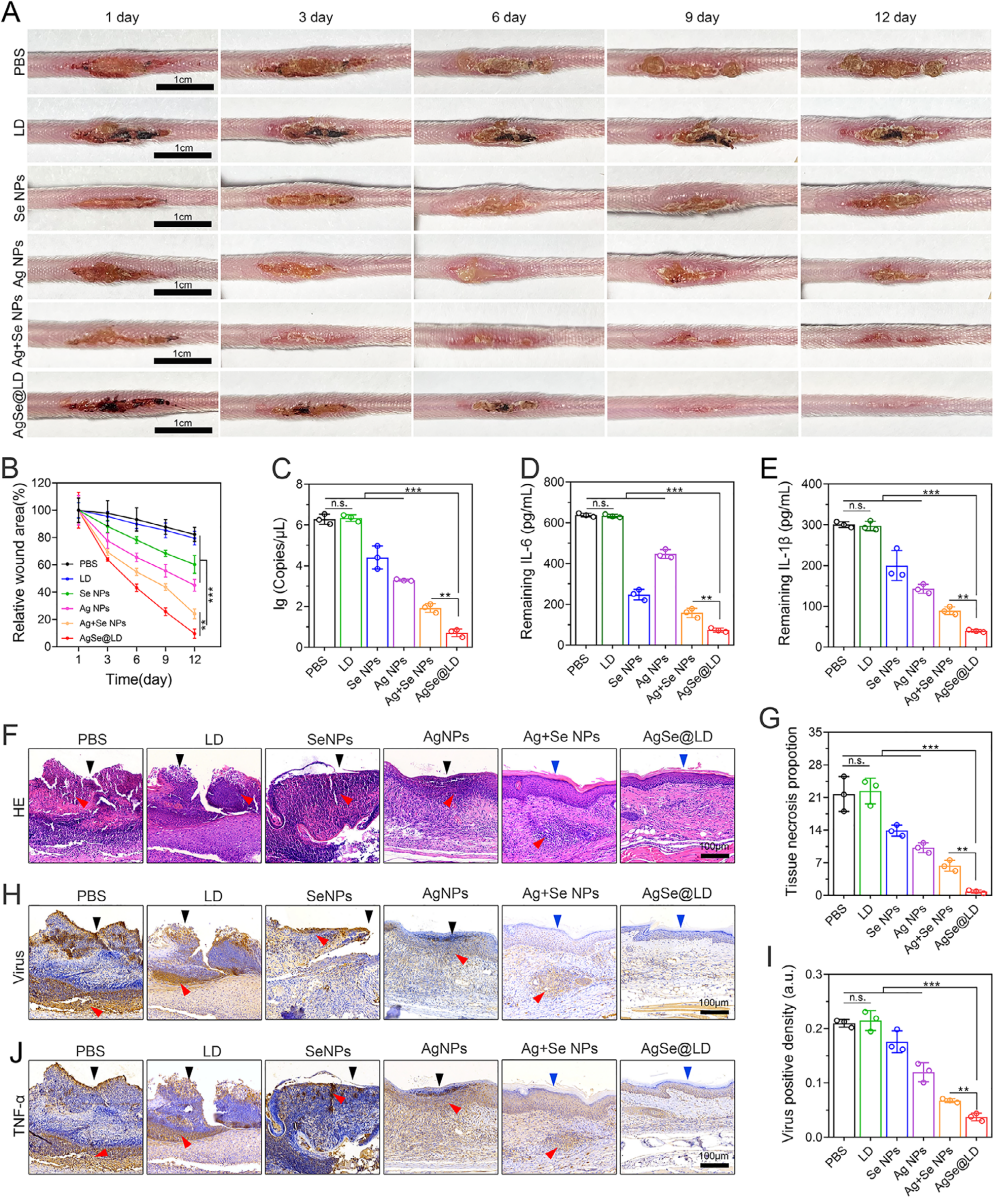
Blocking virus transmission by LD, Se NPs, Ag NPs, Ag+Se NPs and AgSe@LD in vivo. (A) After reinoculation of healthy mouse tails with virus‐containing supernatants derived from mouse tail lesion tissues subjected to different treatments for 7 days, the appearance of wounds was observed (scale bar = 1 cm). (B) Image J software was used for quantitative analysis of tail lesions in the different treatment groups. The data presented are mean ± SD (*n* = 3). (C) Viral loads were detected after reinoculation of healthy mouse tails with viral supernatants. (D, E) Detection of inflammatory factors (IL‐6 and IL‐1β) in the tails of infected mice. The data are presented as the mean ± SD (*n* = 3). (F, G) After reinoculation of healthy mouse tails with virus‐containing supernatants, HE staining of tail lesions and quantitative analysis of necrotic tissue areas of the lesions were performed (scale = 100 µm). (H, I) Immunohistochemical analysis and quantitative analysis of lesion staining intensity after re‐immunisation of healthy mice with viral antigens on the tail (scale = 100 µm). (J) Re‐immunisation of healthy mice was performed at the site of the previous injection, followed by immunohistochemical staining of TNF‐α in the affected area on the tails of the mice. The data are presented as the mean ± SD (*n* = 3). Data were analysed using one‐way analysis of variance. *p < 0.05, **p < 0.01, ***p < 0.001.

After infection with the MPXV, almost 90% of patients develop a rash. The lesions progress from macules (skin lesions with a flat base) to papules (raised, hard, painful skin lesions) to blisters (filled with clear fluid) to pustules (filled with pus), where live viruses are abundant and contribute to transmission [3, 5]. This study aimed to evaluate the effectiveness of Se NPs, Ag NPs and AgSe@LD at blocking virus transmission in vivo to inform prevention strategies. Supernatants from tissue homogenates of lesions in mice treated with various therapeutic groups were used to infect the tails of normal mice through scratches, with results presented in Figure 5A–C. Severe lesions, scabs, erythema and elevated levels of live virus were observed in the PBS and LD groups, suggesting a potential for significant viral transmission to healthy mice. In contrast, the tails of mice in the Se NP and Ag NP groups exhibited moderate damage, with the area of injury decreasing gradually over time and the viral load in the lesions remained at moderate levels. The tails of the Ag+Se NPs and AgSe@LD groups displayed only minor lesions, with a marked reduction in the injured area and a significant decrease in viral load. Notably, the AgSe@LD group demonstrated the most pronounced effect, achieving complete recovery within 12 days. This finding indicates that treatment substantially reduced the viral load in the lesions, likely due to the formation of a stable film that acts as a physical barrier and effectively eradicates live virus within the lesions. These results suggest that AgSe@LD treatment significantly diminishes the presence of live virus in skin lesions of mice, thereby effectively preventing inter‐individual viral transmission. Additionally, an ELISA was conducted to evaluate the levels of inflammatory factors (IL‐6 and IL‐1β) across different treatment groups. As illustrated in Figures 5D,E, the findings were consistent with the in vivo anti‐inflammatory effects observed in all treatment groups. This indicates that both Se NPs and Ag NPs possess inherent anti‐inflammatory activities, which are more pronounced than those of the individual NPs and Ag+Se NPs, likely due to the aggregation effect of AgSe@LD and the resultant higher local concentrations of the therapeutic agents.

Subsequently, HE, Masson and immunohistochemical staining were performed on the collected tail tissues to assess the degree of tissue damage and quantify viral particles and inflammatory factors. We assessed the efficacy of various treatment modalities in preventing or obstructing viral transmission. Following the infusion of normal mice with the focal supernatant, HE staining results (Figures 5F,G and S8) revealed varying degrees of lesions in the different treatment groups. Notably, the Ag+Se NPs and AgSe@LD exhibited milder lesions, with AgSe@LD showing the least degree of tissue damage. Furthermore, immunohistochemical analysis (Figures 5H–J and S7) indicated that the density of viral antigens was significantly higher in the PBS and LD treatment groups, along with stronger nuclear positivity and diffuse expression of TNF‐α and IFN‐γ. In contrast, the groups treated with Ag+Se NPs and AgSe@LD exhibited a marked reduction in both viral antigen density and inflammatory responses, with AgSe@LD displaying the most significant decrease, aligning with the HE staining findings. This enhanced efficacy is likely attributable to the ability of AgSe@LD to form a film upon application to the lesions, thereby increasing the local drug concentration and prolonging its action duration. In summary, the results demonstrated that the tail lesion samples from the AgSe@LD treatment group contained virtually no viable virus, underscoring the potential of AgSe@LD in effectively blocking viral transmission post‐treatment and offering a novel approach for the treatment of MPOX.

### 3.6 Biosafety Evaluation

**FIGURE 6:**
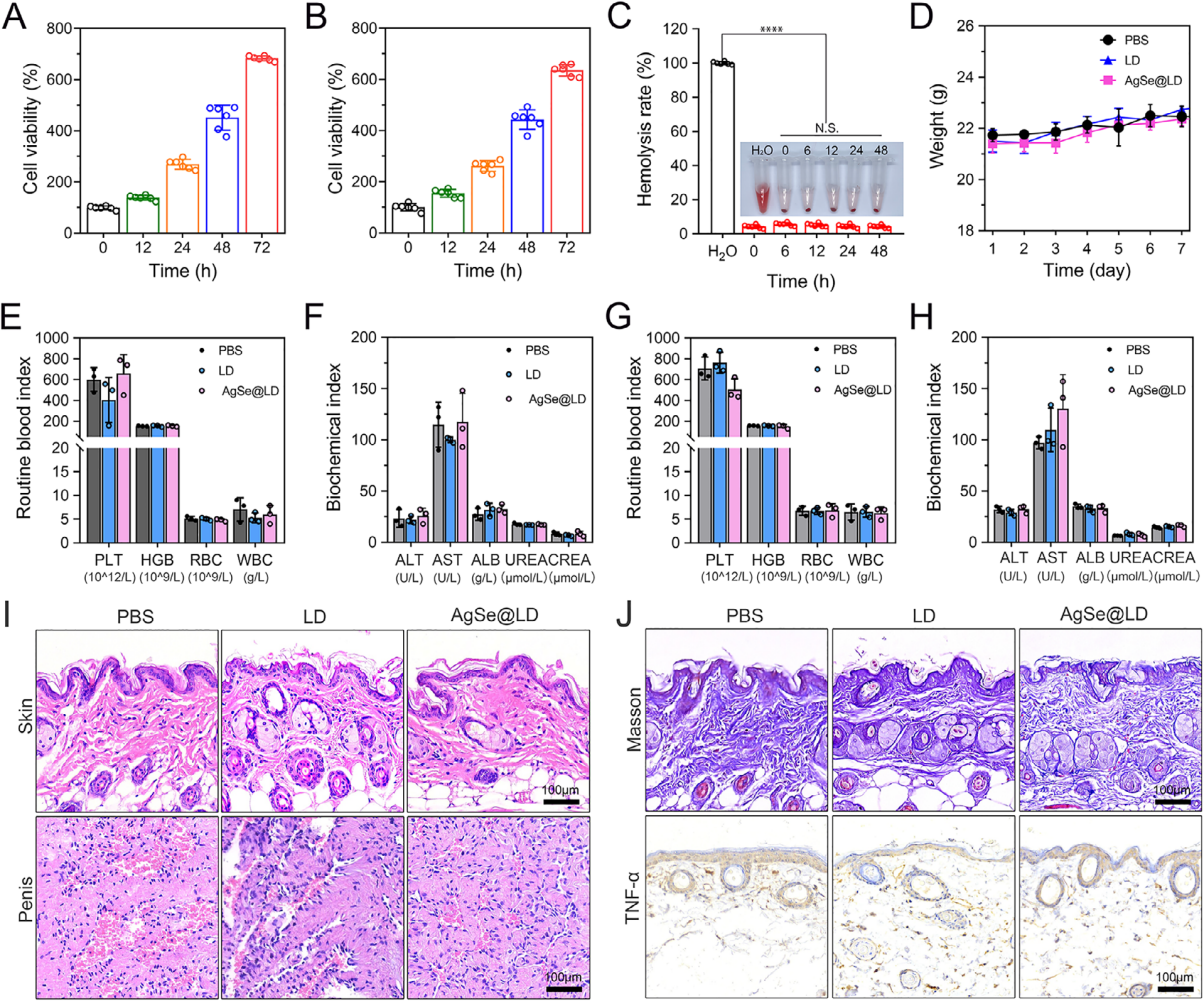
Biocompatibility of AgSe@LD. (A, B) Viability of healthy cells (human umbilical vein endothelial cells and L929 cells) after incubation with AgSe@LD for different times, assessed using Cell Counting Kit‐8. Data indicate the mean ± SD (*n* = 6). (C) Assessment of the lysis of red blood cells incubated with AgSe@LD for different times, showing the mean ± SD (*n* = 6). (D) Weight recording after different dressings were applied to the back skin of mice for different times, with PBS as a control (mean ± SD; *n* = 3). (E, F) Routine blood, liver function and kidney function analyses in mice after applying various dressings to their back skin for 7 days (mean ± SD; *n* = 3). (G, H) Routine blood, liver function and kidney function analyses in mice after different dressings were applied to the mice skin of the perineum for 7 days (mean ± SD; *n* = 3). (I) HE staining of the skin and penis of mice after different dressings were applied to the back or perineum skin for 7 days. (J) Masson's trichrome staining and immunohistochemical analysis of inflammatory cytokines (TNF‐α) after different dressings were applied to the back skin of mice for 7 days. Scale = 100 µm. ns, not significant; *p < 0.05, **p < 0.01, ***p < 0.001.

High biosafety and biocompatibility are critical for the development of new therapeutic agents [61, 62]. In this study, we thoroughly evaluated the in vitro and in vivo biocompatibility of Ag NPs, Se NPs and AgSe@LD. We first evaluated the cytotoxicity of Ag NPs and Se NPs at different concentrations (Figure S9). Ag NPs showed no cytotoxic effect even at high concentrations, whereas Se NPs showed a slight decrease in cell survival with increasing concentration. Consequently, we selected optimal concentrations of both nanoparticles for the preparation of AgSe@LD. As illustrated in Figure 6A,B, AgSe@LD was co‐incubated with cells for 72 h, with cell viability assessed at 0, 12, 24, 48 and 72 h. No significant inhibitory effects on the proliferation of normal cells were detected, indicating that AgSe@LD possesses low cytotoxicity. Additionally, haemolysis assays conducted at 0, 6, 12, 24 and 48 h revealed that exposure to AgSe@LD at specific concentrations did not induce haemolysis (Figure 6C), confirming its low haemolytic activity.

To evaluate in *vivo* compatibility, AgSe@LD (200 µL, 30 mg/kg) was administered to the dorsal region of mice. No notable differences in body weight were observed between the PBS‐treated and experimental groups (Figure 6D), suggesting the absence of systemic adverse effects. Standard blood tests, including assessments of red blood cells, haemoglobin, white blood cells and platelets, demonstrated no significant differences between the AgSe@LD and PBS‐treated groups (Figure 6E). Furthermore, evaluations of plasma biochemical markers related to liver function—such as ALB, AST and ALT—as well as renal function markers, including creatinine (CR) and BUN, indicated that levels in the AgSe@LD‐treated group remained unchanged compared to the PBS treatment group (Figure 6F). These results suggest that AgSe@LD does not induce haematological disorders or compromise liver and kidney function. Additionally, AgSe@LD was applied to mice to assess potential genital toxicity. Comparison of routine blood tests and plasma biochemical indicators of liver and kidney function revealed no significant abnormalities compared to the PBS treatment group (Figure 6G,H). Histological evaluations through HE and Masson staining of the skin and genital region after 7 days of exposure to a high concentration of AgSe@LD (3 mg/mL) revealed no significant tissue damage or inflammation (Figure 6I,J). This indicated that AgSe@LD was safe for normal skin.

It is worth noting that MPOX skin lesions are typically characterised by multi‐site, multifocal and scattered distribution, which raises higher safety and practicality requirements for the application of AgSe@LD, especially regarding potential Ag and Se poisoning from large‐area application on the lesions. To evaluate whether AgSe@LD can safely and effectively cover multiple lesions and considering that MPOX lesions are mostly small pustules, we simulated multiple MPOX lesions and assessed safety concerns related to large‐area damage by removing large and multiple areas of skin on the backs of mice. As shown in Figure S10, after applying LD and AgSe@LD to the large injured areas for three consecutive days, no significant redness, swelling, or inflammation was observed in the lesions of mice in either group compared to the control group, indicating that AgSe@LD does not exacerbate skin damage. Furthermore, no significant weight loss or changes in activity were observed in the mice (Figure S11A). We collected blood, skin and major organs from the mice and no significant alterations were observed in the complete blood count or blood biochemistry among the three groups (Figures S11B,C). Histopathological examination of the lesions and major organs (heart, liver, spleen, lungs and kidneys) via H&E staining also showed no significant differences (Figures S11D,E). Additionally, we collected the lesions for further analysis. These results demonstrate that AgSe@LD is safe for treating large‐area injuries caused by multiple mpox lesions and does not induce Ag or Se poisoning.

In summary, AgSe@LD demonstrates low cytotoxicity and does not exhibit adverse effects on blood cells or the hematopoietic system, nor does it induce Ag and Se poisoning at the lesion site. Thus, AgSe@LD exhibits excellent biocompatibility when applied to the dorsal skin and genital area of mice, confirming its potential as a safe and valuable biomaterial for therapeutic applications.

## 4 Discussion and Conclusion

The global resurgence of monkeypox (MPOX) has underscored an urgent need for effective therapeutic and preventive strategies, particularly those capable of mitigating viral transmission through skin lesions. In this study, we developed a novel nano‐silver‐selenium liquid dressing (AgSe@LD) by incorporating Ag NPs and Se NPs into a commercially available liquid dressing. Our results demonstrate that AgSe@LD not only exhibits potent antiviral and anti‐inflammatory activities but also effectively blocks viral transmission, thereby addressing two critical aspects of MPOX treatment and prevention.

The innovation of this study lies in the synergistic combination of Ag NPs and Se NPs, which target multiple stages of the viral life cycle through complementary mechanisms. While Ag NPs are well‐known for their broad‐spectrum antiviral properties through mechanisms such as viral membrane disruption, ROS generation and inhibition of viral replication, Se NPs contribute through antioxidant, anti‐inflammatory and immunomodulatory effects. To our knowledge, this is the first report to combine these two nanoparticles specifically for the treatment of Orthopoxvirus infections, representing a significant advancement in nanomaterial‐based antiviral therapeutics. Another key innovation is the formulation of AgSe@LD as a liquid dressing that forms a stable, adhesive and wash‐resistant film over lesions. This physical barrier not only prolongs the local retention and action of the nanoparticles but also prevents environmental contamination and person‐to‐person transmission—a major route of MPXV spread. The dressing's resistance to water, alcohol and soap further enhances its practicality for real‐world use, particularly in resource‐limited settings.

Our in vitro and in vivo results consistently showed that AgSe@LD outperformed individual NPs or their simple mixture (Ag+Se NPs), highlighting the importance of the formulated composite. The significant reduction in viral load, inflammatory cytokines and tissue damage, coupled with accelerated wound healing, underscores the multifunctional capability of AgSe@LD. Moreover, the transmission‐blocking experiments provide compelling evidence that AgSe@LD can effectively reduce the risk of secondary infections, a feature rarely addressed by conventional antivirals. The excellent biocompatibility and biosafety profile of AgSe@LD further support its translational potential. Unlike some nanoparticle‐based therapies that pose toxicity concerns, AgSe@LD showed no significant cytotoxicity, haemolytic activity, or systemic toxicity in mice, even upon prolonged exposure. This safety, combined with the use of commercially available liquid dressings, positions AgSe@LD as a cost‐effective and scalable solution for MPOX management.

In conclusion, AgSe@LD represents a pioneering approach in the fight against MPOX, integrating antiviral therapy with transmission prevention in a single, user‐friendly formulation. Its dual functionality, robust performance and high safety margin make it a promising candidate for clinical translation. Future studies should focus on optimising the formulation for human use, evaluating its efficacy against other poxviruses and exploring its potential in combination with systemic antivirals or vaccines.

## Author Contributions

**Wei Wang**: conceptualisation, visualisation, funding acquisition, writing – original draft. **Mengjun Li**: conceptualisation, visualisation, writing – original draft. **Zining Liu**: methodology, visualisation. **Jiayin Chen**: methodology, investigation, visualisation. **Ke Liu**: methodology, visualisation. **Fengfang Wei**: visualisation. **Junrui Li**: methodology. **Yixuan Xie**: investigation. **Yushan Jiang**: investigation. **Tyuji Hoshin**: methodology, visualisation. **Vladislav Victorovich Khrustalev**: methodology, visualisation. **Minghui Yang**: writing – review and editing. **Hua Ma**: supervision, writing – review and editing. **Rulin Zhang**: project administration, writing – review and editing. **Chenguang Shen**: funding acquisition, project administration, writing – review and editing. **Yuhui Liao**: funding acquisition, project administration writing – review and editing.

## Ethics Statement

All experiments involving animals were reviewed and approved by the Experimental Animal Ethics Committee of the Experimental Animal Centre of Southern Medical University (D202310‐1). All human skin adhesion studies were reviewed and approved by the Ethics Committee of Kunming Medical University (Approval No.: KMMU2024MEC287). All participating patients were informed and written informed consent was obtained from all participants. All procedures involving human subjects were conducted in accordance with the principles of the Declaration of Helsinki.

## Conflicts of Interest

The authors declare no conflicts of interest.

## Supporting information




**Supporting File**: exp270183‐sup‐0001‐SuppMat.docx.

## Data Availability

The data in this work are available in the manuscript or supplementary information or available from the corresponding author upon reasonable request.
