# Supplementary material for: Nano‐Silver‐Selenium Liquid Dressing Facilitates Treatment of Monkeypox and Prevention of Viral Transmission in a Surrogate Mouse Model
Source: Exploration (Beijing). 2026 May 28;6(3):20240253. doi: 10.1002/EXP.20240253 (PMC13317732; doi:10.1002/EXP.20240253)
Supplement: Supplementary file 1 — Supporting File: exp270183‐sup‐0001‐SuppMat.docx. [file EXP2-6-20240253-s001.docx]

Nano-Silver-Selenium Liquid Dressing Facilitates Treatment of Monkeypox and Prevention of Viral Transmission in a Surrogate Mouse Model

Wei Wang^1,2‡^, Mengjun Li^2,3‡^, Zining Liu^2‡^, Jiayin Chen^3‡^, Ke Liu^3^, Fengfang Wei^4^,Junrui Li^1^, Yixuan Xie^5^, Yushan Jiang^3^, Tyuji Hoshino ^6^, Vladislav Victorovich Khrustalev ^7^, Hua Ma^1^*, Ruilin Zhang^1^*, Chenguang She^2,3^*, Yuhui Liao^1^*

^1^Institute for Engineering Medicine, NHC Key Laboratory of Drug Addiction Medicine, Kunming Medical University, Kunming 650500, China.

^2^ BSL-3 Laboratory (Guangdong), Guangdong Provincial Key Laboratory of Tropical Disease Research, School of Public Health, Department of Laboratory Medicine, Zhujiang Hospital, Southern Medical University, Guangzhou 510515, Guangdong, China.

^3^ Key Laboratory of Infectious Diseases Research in South China (Southern Medical University), Ministry of Education, Guangzhou 510515, Guangdong, China.

^4^ The Affiliated Hospital of Putian University, Putian Children's Hospital, 351100, Putian, China.

^5^ Hainan Medical College Affiliated Danzhou People's Hospital, Danzhou 571700, Hainan, China.

^6^ Graduate School of Pharmaceutical Sciences, Chiba University, Inohana 1-8-1, Chuo-ku, Chiba 260-8675, Japan.

^7^ Department of General Chemistry, Belarusian State Medical University, Dzerzhinskogo 83, Minsk, 220045, Belarus.

‡These authors contributed equally to this work.

^*^Correspondence: mahua1009@139.com (H. Ma); zhangruilin@kmmu.edu.cn (R. Lin); a124965468@smu.edu.cn (C. Shen); liaoyh8@mail.sysu.edu.cn (Y. Liao)


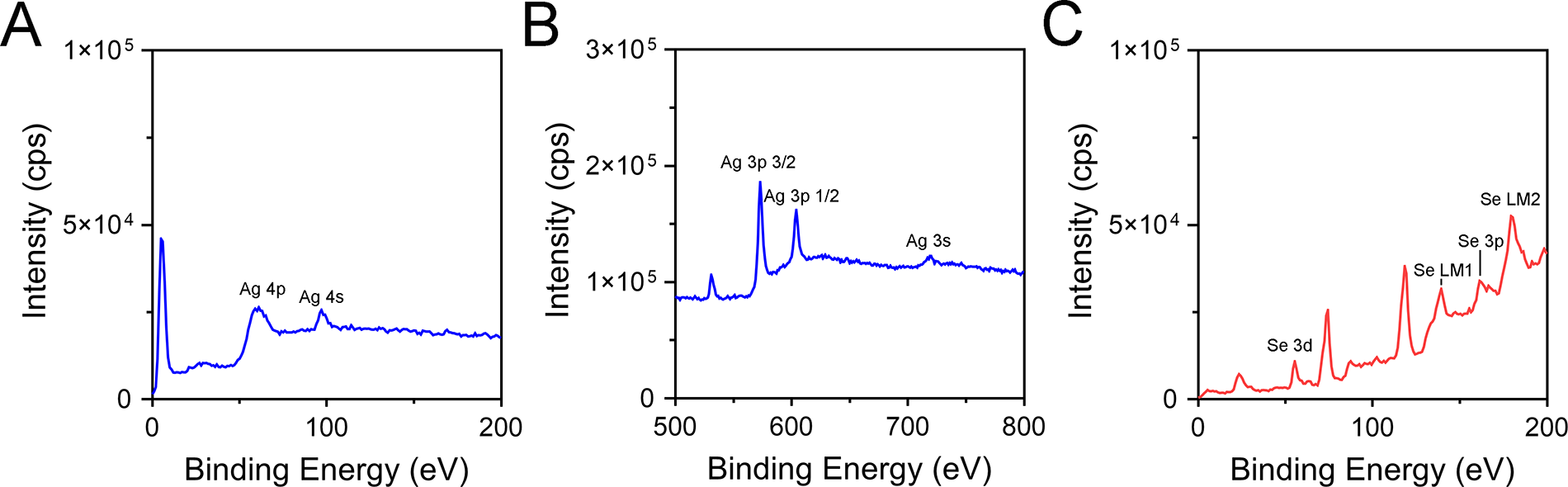


**Figure S1.** The local magnified image of X-ray photoelectron spectroscopy (XPS) analysis was shown in Figure 1A. (A-B) XPS analysis of Ag NPs at 0-200 binding energies (A) and 500-800 binding energies (B). (C) XPS analysis of Se NPs at 0-200 binding energies.


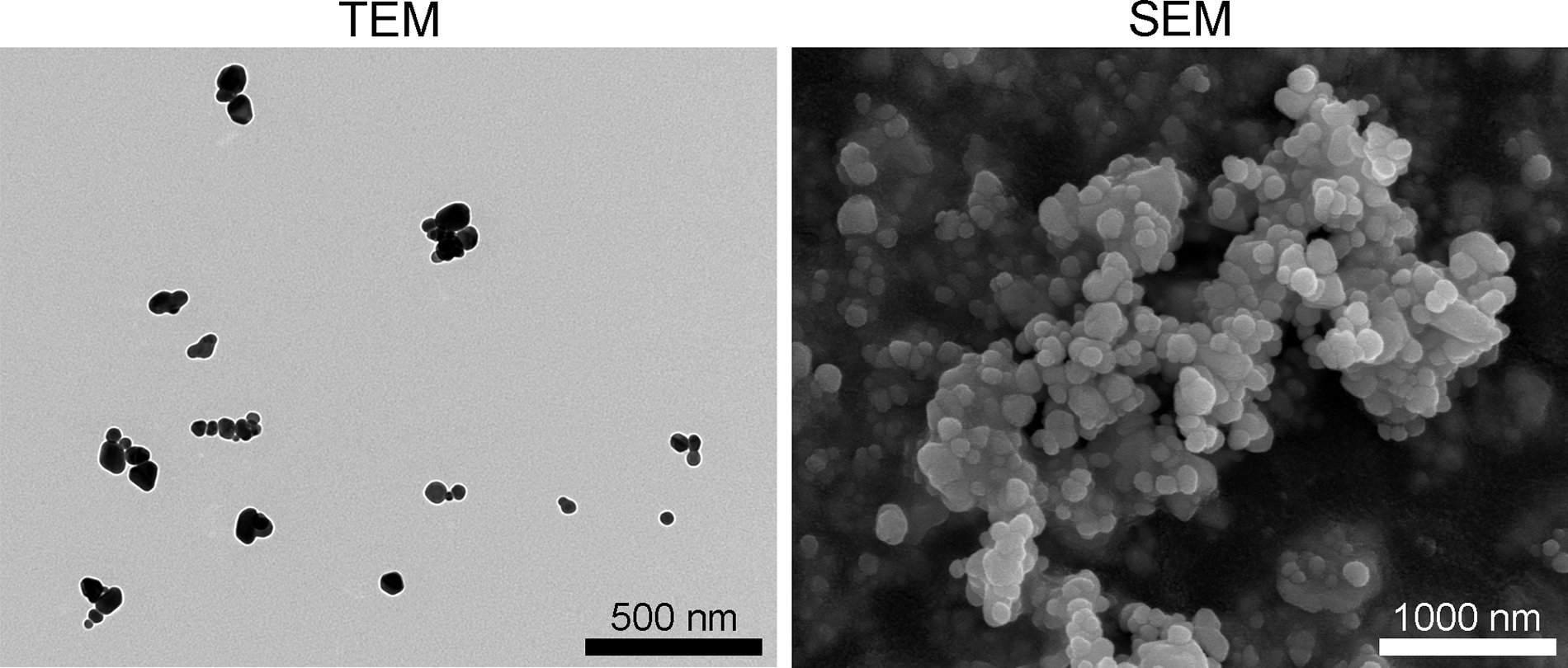


**Figure S2.** Transmission electron microscope (TEM) and scanning electron microscope (SEM) images of AgNPs.


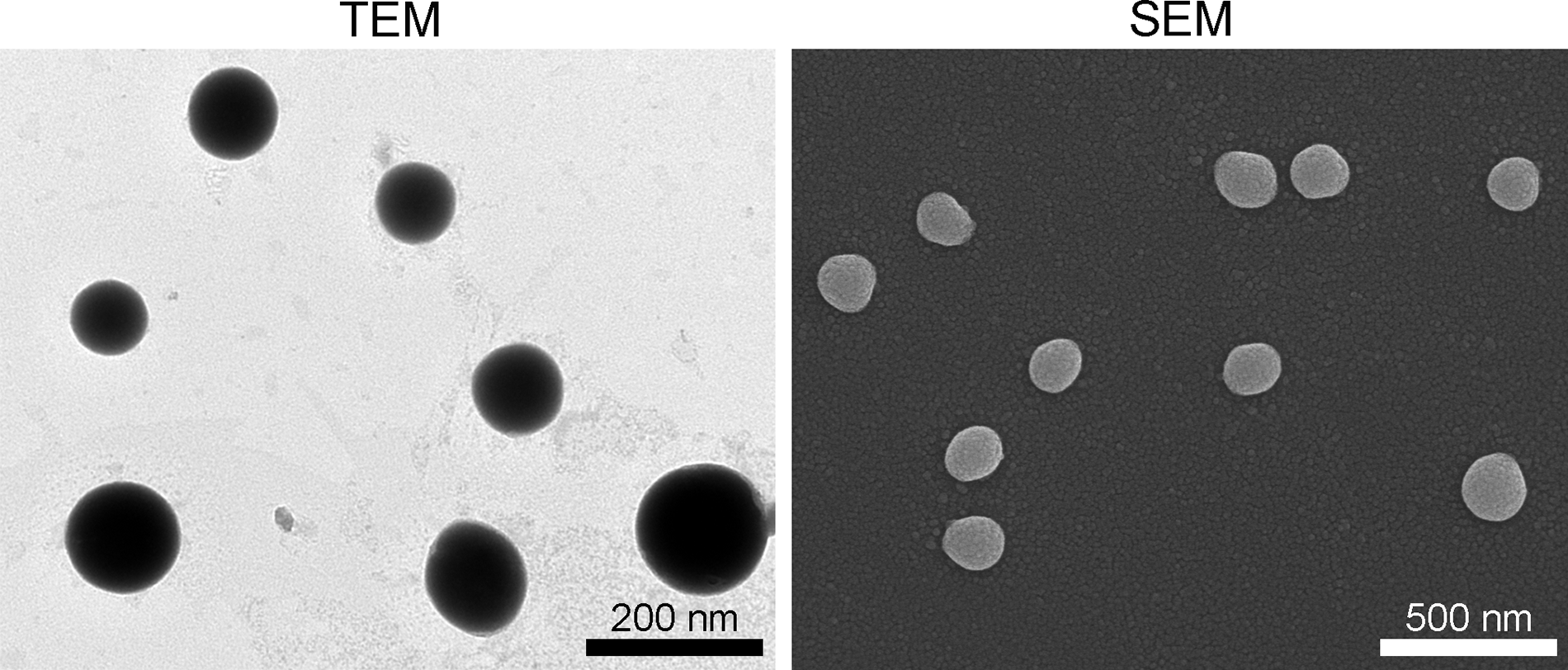


**Figure S3.** Transmission electron microscope (TEM) and scanning electron microscope (SEM) images of Se NPs.


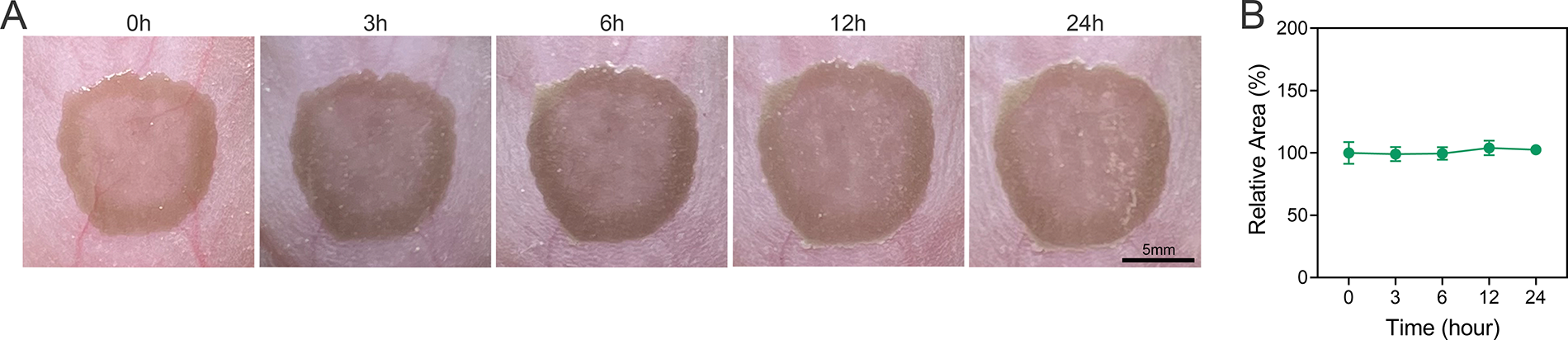


**Figure S4.** The adhesion images (A) and statistical analysis (B) of AgSe@LD on the back skin of mice in different time. scale bar =5 mm.


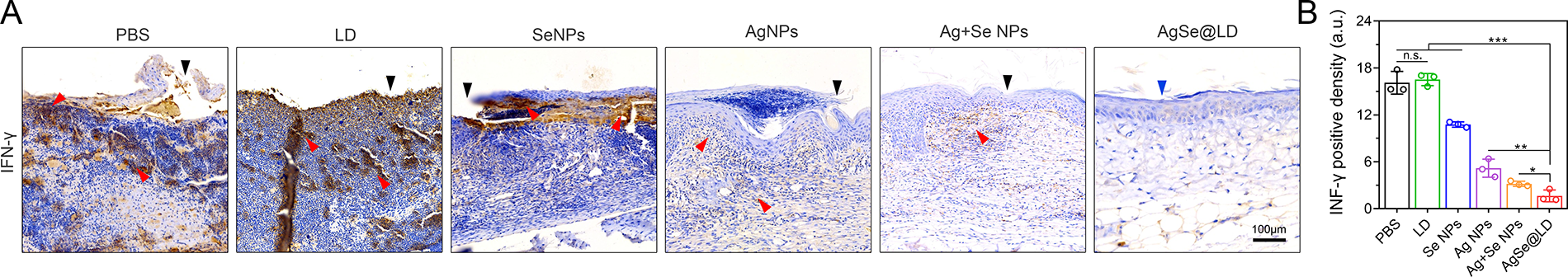


**Figure S5.** Immunohistochemical staining for INF-γ (A) and statistical analysis (B) of staining intensity in tail lesions subjected to different treatments (scale bars = 100 µm). Data are expressed as the mean ± SD (n = 3). (*, P < 0.05; **, P < 0.01; ***, P < 0.001).


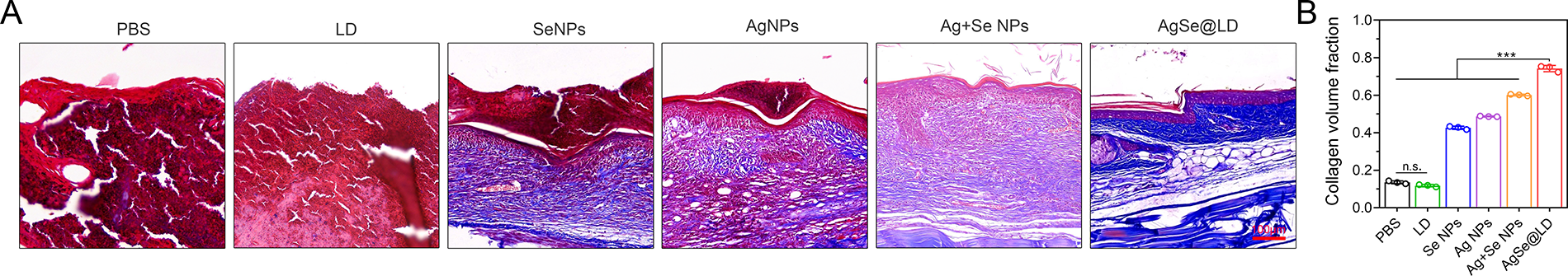


**Figure S6.** Masson staining (A) and statistical analysis (B) of staining intensity in tail lesions subjected to different treatments (scale bars = 100 µm). Data are expressed as the mean ± SD (n = 3). (*, P < 0.05; **, P < 0.01; ***, P < 0.001).


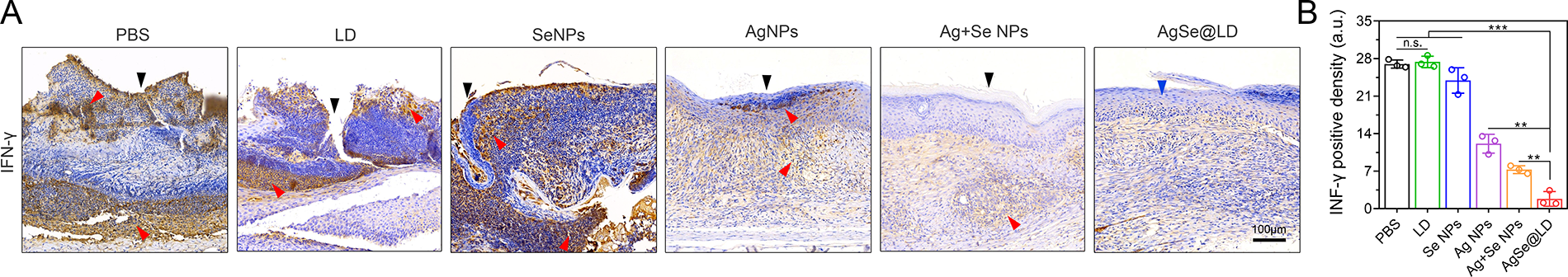


**Figure S7.** Immunohistochemical staining for INF-γ (A) and statistical analysis (B) of staining intensity in blocking virus transmission (scale bars = 100 µm). Data are expressed as the mean ± SD (n = 3). (*, P < 0.05; **, P < 0.01; ***, P < 0.001).


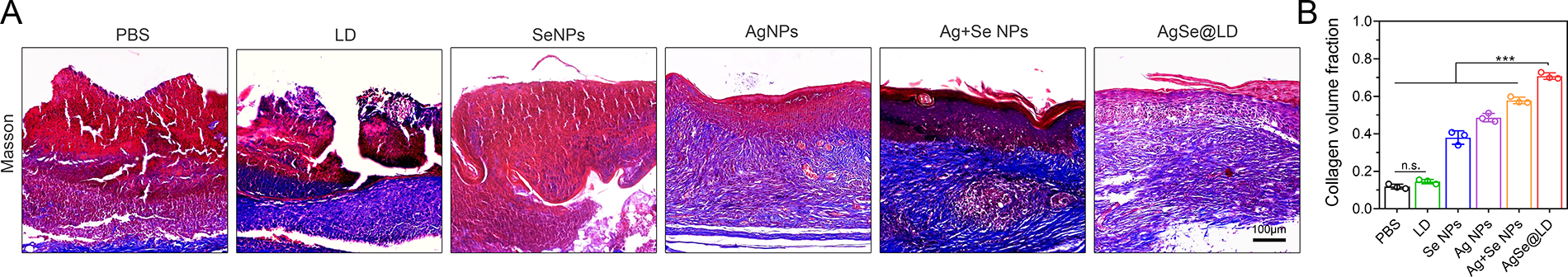


**Figure S8.** Masson staining (A) and statistical analysis (B) of staining intensity in in blocking virus transmission (scale bars = 100 µm). Data are expressed as the mean ± SD (n = 3). (*, P < 0.05; **, P < 0.01; ***, P < 0.001).


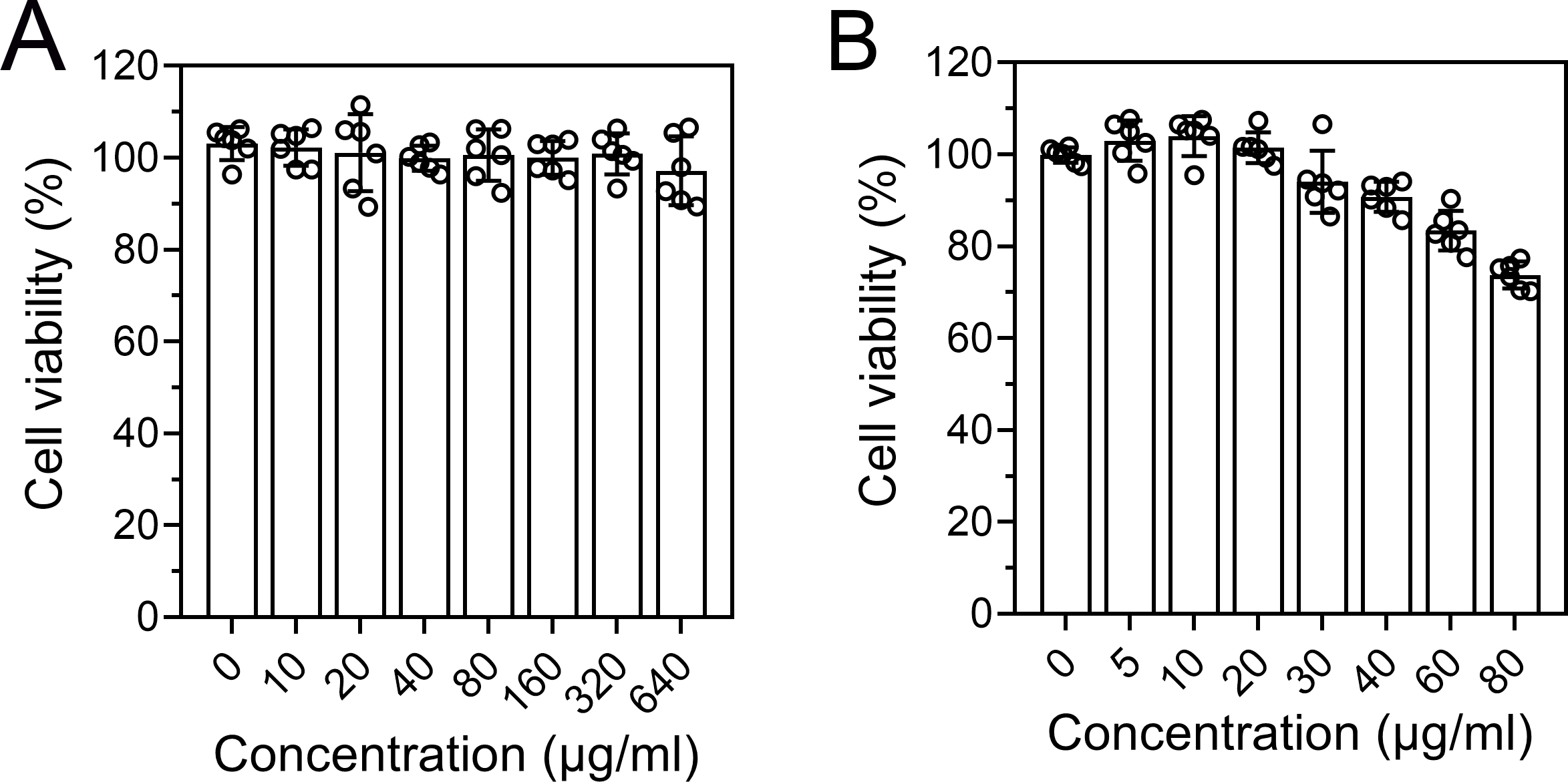


**Figure S9.** **The cytotoxic effect of Ag NPs and Se NPs on normal cells.** (A–B) Viability of healthy cells (human umbilical vein endothelial cells and L929 cells) after incubation with Ag NPs (A) and Se NPs (B) for different concentrations, assessed using Cell Counting Kit-8. Data indicate the mean ± SD (n = 6).


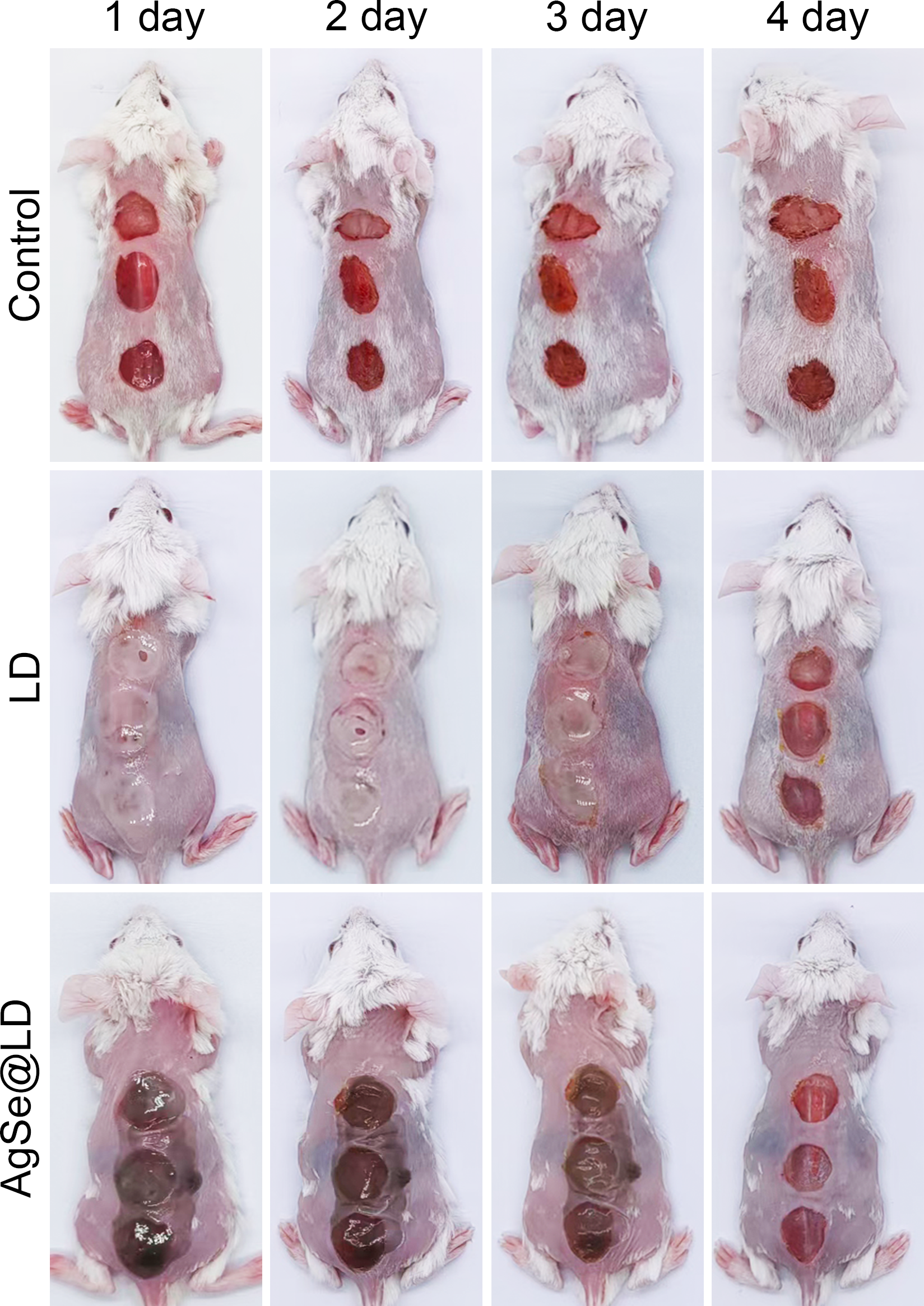


**Figure S10.** Photographs of LD and AgSe@LD coating on multiple and extensive lesions in mice.


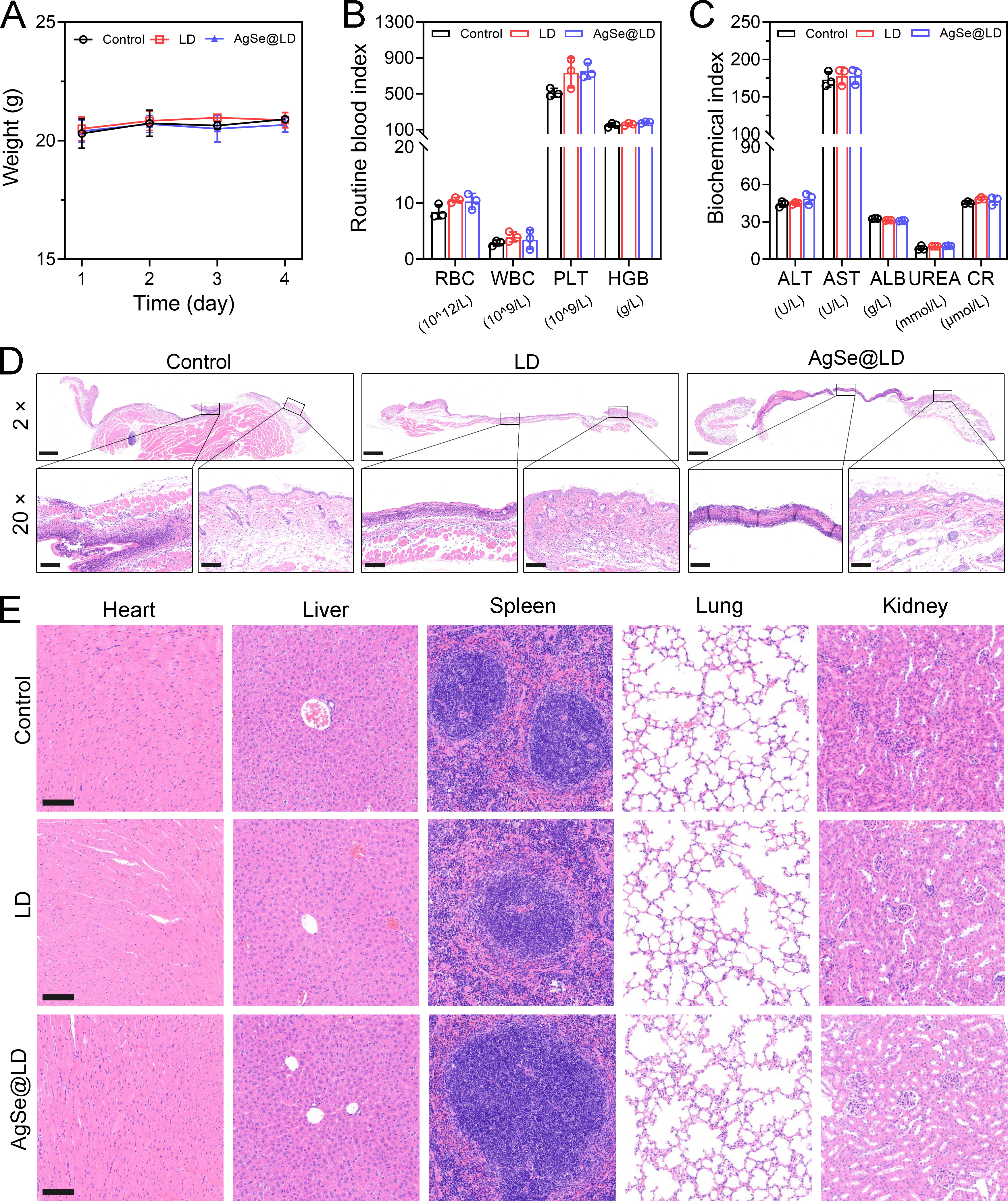


**Figure S11.** **Safety of AgSe@LD coating on multiple and extensive lesions in mice.** (A) Weight recording after different dressings were applied to the backs skin of mice for different times. (mean ± SD; *n* = 3). (B–C) Routine blood, liver function, and kidney function analyses in mice after applying various dressings to multiple and extensive lesions for 3days (mean ± SD; *n* = 3). (D-E) HE staining of the skin (2 × and 20 ×) and main organs (20 ×)after different dressings were applied to multiple and extensive lesions for 3days. 2 ×, Scale = 500 μm. 20 ×, Scale = 100 μm.
